# Ethical and legal implications of implementing risk algorithms for early detection and screening for oesophageal cancer, now and in the future

**DOI:** 10.1371/journal.pone.0293576

**Published:** 2023-10-30

**Authors:** Tanya Brigden, Colin Mitchell, Elizabeth Redrup Hill, Alison Hall

**Affiliations:** PHG Foundation, University of Cambridge, Cambridge, United Kingdom; Deakin University, AUSTRALIA

## Abstract

**Background:**

Oesophageal cancer has significant morbidity and mortality but late diagnosis is common since early signs of disease are frequently misinterpreted. Project DELTA aims to enable earlier detection and treatment through targeted screening using a novel risk prediction algorithm for oesophageal cancer (incorporating risk factors of Barrett’s oesophagus including prescriptions for acid-reducing medications (CanPredict)), together with a non-invasive, low-cost sampling device (Cytosponge^TM^). However, there are many barriers to implementation, and this paper identifies key ethical and legal challenges to implementing these personalised prevention strategies for Barrett’s oesophagus/oesophageal cancer.

**Methods:**

To identify ethical and legal issues relevant to the deployment of a risk prediction tool for oesophageal cancer into primary care, we adopted an interdisciplinary approach, incorporating targeted informal literature reviews, interviews with expert collaborators, a multidisciplinary workshop and ethical and legal analysis.

**Results:**

Successful implementation raises many issues including ensuring transparency and effective risk communication; addressing bias and inequity; managing resources appropriately and avoiding exceptionalism. Clinicians will need support and training to use cancer risk prediction algorithms, ensuring that they understand how risk algorithms supplement rather than replace medical decision-making. Workshop participants had concerns about liability for harms arising from risk algorithms, including from potential bias and inequitable implementation. Determining strategies for risk communication enabling transparency but avoiding exceptionalist approaches are a significant challenge. Future challenges include using artificial intelligence to bolster risk assessment, incorporating genomics into risk tools, and deployment by non-health professional users. However, these strategies could improve detection and outcomes.

**Conclusions:**

Novel pathways incorporating risk prediction algorithms hold considerable promise, especially when combined with low-cost sampling. However immediate priorities should be to develop risk communication strategies that take account of using validated risk algorithms, and to ensure equitable implementation. Resolving questions about liability for harms arising should be a longer-term objective.

## 1. Introduction

Over the last two decades, there has been a shift in healthcare from primarily operating as a service treating and managing extant disease, to one which also aims to identify those at highest risk of developing disease and pre-emptively intervene to mitigate or prevent that disease occurring [1]. This change from an opportunistic phenotype driven service focused on treatment and cure, to a data driven service committed to prevention remains somewhat aspirational. However, it has been facilitated by two significant developments–the burgeoning knowledge of how genetic and genomic changes contribute to disease, and the use of statistical mechanisms and tools to integrate and analyse data, both at the level of the individual and the population [2, 3]. Risk stratification, through the use of a risk prediction algorithm, is one means of ensuring that those at greatest risk of developing disease have earlier access to appropriate interventions and risk management [4]. One example is provided by the development of a risk prediction algorithm for identifying patients at increased risk of oesophageal cancer, the ‘CanPredict oesophageal cancer algorithm’ (CanPredict) [5]. This risk algorithm has been developed for application within a primary care pathway, using electronic health records (EHRs) held within primary care to identify those at highest risk of developing oesophageal cancer. By offering a non-invasive test (Cytosponge^TM^) to those at highest risk, cellular oesophageal samples could be obtained and analysed at scale using novel staining and digital pathology to identify those with Barrett’s oesophagus, a precursor for the future development of oesophageal cancer [6].

### 1.1 Barrett’s oesophagus: Novel opportunities for personalised prevention

Barrett’s oesophagus is characterised by intestinal metaplasia, a process in which the normal cellular lining of the lower oesophagus made up of stratified squamous epithelial cells is replaced by columnar epithelium [7, 8]. These cellular changes result from gastro-oesophageal reflux disease (GORD) in which acid and bile from the stomach pass into the lower part of the oesophagus causing symptoms of heartburn. This is a very common disorder, and the global prevalence is estimated to be between 3–14%, with prevalence broadly resembling the incidence of gastro-oesophageal reflux [9]. There is debate about the diagnostic criteria for Barrett’s oesophagus and its pathogenesis [10]. Different patterns of Barrett’s oesophagus progression have been observed, characterised by alternative forms of clonal expansion and evolution. However, most cases of Barrett’s oesophagus will not progress to oesophageal adenocarcinoma and of those with the lowest-risk (non-dysplastic Barrett’s oesophagus), only 0.3% per annum have a risk of progression to oesophageal adenocarcinoma [11, 12]. Due to its non-specific symptoms of heartburn, and difficulty swallowing, this cancer is often diagnosed late, resulting in a poor five year survival rate (around 18% for women and 16% for men), ranking in the worst four or five cancer sites in England [13]. Predicted 10-year net survival in England is particularly low for men (12.5%) [14]. Therefore, the early detection of Barrett’s oesophagus provides a significant opportunity for improving population health.

### 1.2 Cancer risk prediction tools

Cancer risk prediction tools are statistical formulae that calculate the probability that a patient will develop a cancer within a specified period taking account of personal characteristics and medical data. Cancer referral decision-making could be aided by the use of cancer risk algorithms and used to support the primary care physician’s decisions about the significance of the presenting symptoms, and onward referral for investigation or specialist assessment. This potential is particularly relevant in England where, despite considerable investment being put into improving early diagnosis, mortality figures are poor in relation to other developed countries [15, 16]. NICE guidance advises General Practitioners (GPs) to use the 2-week-wait referral pathway if they suspect cancer [17]; the patient is then seen by a specialist within a target of two weeks. When patients present with symptoms to primary care, GPs must judge the individual patient’s likelihood of cancer, however discriminating between patients who should be referred on the 2-week-wait pathway from those who do not need to is difficult, especially where early cancers present with vague, non-specific symptoms that could easily be attributed to other conditions [18].

However, cancer risk prediction tools remain an underused resource within primary care in England despite being integrated in the majority of primary care computer systems [19]. Qualitative studies have identified a number of barriers and facilitators to the implementation of cancer risk calculators/prediction tools in clinical practice, ranging from GP preparedness to use (and rely on) the risk tool, to more practical challenges around availability and integrating them into the clinical workflow [20, 21]. Integration into electronic health record systems could facilitate their use and some cancer risk assessment tools, most notably QCancer® and Risk-Assessment Tools (RATs), have been integrated with the electronic health record in some parts of UK primary care [20, 22].

### 1.3 Development of a risk algorithm for oesophageal cancer

Project DELTA aims to develop a novel risk prediction algorithm for oesophageal cancer which could be utilised within primary care [5]. Primary care electronic records contain a rich diversity of demographic, clinical and medication information which could be used for prediction and prevention [23]. Professor Hippisley-Cox and her team have developed a range of risk prediction algorithms, most notably QRISK for cardiovascular disease fifteen years ago, which is recommended by NICE [24] and which is now integrated into the NHS Health Check programme and regularly updated as data quality improves and requirements evolve [25]. Risk prediction algorithms have also been developed more recently in the context of novel infectious diseases, to identify vulnerable patients at greater risk of morbidity and mortality from COVID-19 (QCovid) who were prioritised for interventions including early access to vaccination and advice and support for shielding [26].

However, there is no currently available tool which is able to collate relevant demographic, clinical and medical factors derived from primary care electronic records to estimate the 10-year risk of developing oesophageal cancer. In order to develop this risk prediction algorithm, the researchers used the QResearch methodology adopted for other risk algorithms. They utilised a master anonymised dataset derived from a coalition of GP practices which form part of the QResearch network containing records from around 30 million patients. The methods used for development of the novel risk prediction algorithm for oesophageal cancer are described more fully in a recent publication [5]. A range of risk model variables were selected comprising established risk factors and some novel risk factors. Established factors derived from literature review and expert opinion included demographic and lifestyle factors such as age, ethnicity, deprivation, body mass index (BMI) smoking, alcohol, family history and a range of medical conditions including a medical history of Barrett’s oesophagus and/or bowel cancer, prior lung or blood cancer and hiatus hernia. Deidentified individuals who have a family history of relevant disease were excluded from the analysis and missing values imputed using prespecified methods [5].

A novel element of this work was the inclusion of certain types of medication as relevant risk factors, including prescriptions for proton pump inhibitors, and H2 blockers which both reduce the amount of stomach acid made by glands lining the stomach in different ways. Proton pump inhibitors (PPIs) prevent the parietal cells lining of the stomach from producing acid resulting in less acid being produced to digest food [27]. H2 receptor blocker medications also work by reducing the amount of stomach acid released by these glands. Both types of drug are used to relieve the symptoms of gastroesophageal reflux disease and are commonly prescribed within primary care [28]. However H2 receptor blocker medications such as famotidine and nizatidine and PPIs such as omeprazole and esomeprazole are also available over the counter [29].

The predictors in the final algorithms were age, BMI, smoking, alcohol, ethnicity, Barrett’s oesophagus, hiatus hernia, *H*. *pylori* infection, use of PPIs, anaemia, lung and blood cancer (together with breast cancer in women) [5]. Since the disease manifests differently in men and women, the final risk model was fitted separately in men and women and validated using two separate sets of data. The final risk model shows good levels of calibration and discrimination and explains over 57% of the variation in 10-year risk of oesophageal cancer with improved sensitivity and specificity compared to existing risk prediction algorithms. If these algorithms were fully implemented, Hippisley-Cox and colleagues estimate that using them would result in improvements in cancer detection, since identifying the top 25% of patients at highest risk would capture 76% of oesophageal cancers that would develop over the next 10 years [5].

Given these potential benefits to population health, and the current relatively low uptake of cancer risk algorithms in England, we aimed to identify and evaluate the ethical and legal challenges and opportunities in implementing risk algorithms for early detection and screening of oesophageal cancer in primary care. We identify a set of ethical, legal and associated practical issues which we discuss in our Results. However, this is a rapidly developing and highly dynamic space and it is likely that at least some novel or additional challenges will arise in the near future as, for example, new AI-driven techniques are adopted or additional and complex risk factors such as genomic risks are included in risk prediction. We discuss how key potential developments may generate additional considerations and how they may addressed in our Discussion below.

## 2. Materials and methods

We adopted an interdisciplinary approach to identifying ethical and legal challenges and opportunities as part of a collaborative project which brought together academics, technology developers, patient and disease groups, a policy think tank and an academic health sciences network (Project DELTA). Our research involved several components: an informal, targeted review of the literature; two expert interviews with DELTA collaborators; a multidisciplinary workshop with key stakeholders; and a process of legal and ethical analysis to extrapolate key themes and findings.

The initial phase of research comprised a detailed but unstructured review of existing legal, regulatory and philosophical sources using search engines including Ovid Medline/PubMed, to understand the potential ethical and legal challenges raised by the use of algorithmic tools for stratification. This was supplemented by searches for relevant policy and grey literature in the public domain. Searches used combinations of free text terms including “Barrett’s Oesophagus”, “Oesophageal cancer”, “cancer risk prediction algorithm”, “cancer risk assessment tool”, “primary care”, “cancer decision support tool”, “risk stratification”, “ethics”, “implication”, and “risk communication”. The reference lists of literature identified through these searches were also reviewed. Our aim was to evaluate how these ethical, legal and regulatory considerations potentially impacted on the development of risk algorithms for oesophageal cancer, to understand the challenges that might be encountered during the research phase, and to explore the potential barriers to implementing a risk algorithm within primary care, to identify those at greatest risk of developing Barrett’s oesophagus, and subsequent oesophageal cancer.

To supplement this desk based research and as part of the workshop development process, we interviewed an expert developing a risk tool for Barrett’s oesophagus, Professor Julia Hippisley-Cox, to better understand the inputs for the model and the range of potential applications in healthcare. We also interviewed a key researcher working on the clinical implementation of the Cytosponge^TM^ test, Professor Rebecca Fitzgerald, to clarify how those identified at high risk by the risk tool might be referred for subsequent investigation using Cytosponge^TM.^ These two experts were purposively recruited through having leadership roles within the Project DELTA collaboration, namely the Principal Investigator and the senior researcher leading the development of the risk algorithm for oesophageal cancer. Formal consents were not sought for these interviews as the interviewees were existing collaborators in an externally funded project, with closely aligned research objectives.

A second round of preparatory work was undertaken, using the information generated from these expert interviews, together with the learning from unstructured literature searches, to develop a comprehensive list of potential use cases for the risk-prediction algorithm. As part of the workshop development process, we used these exemplars to develop a preliminary list of potential ethical and legal/regulatory challenges that might arise. We then tested these preliminary findings with our two experts in order to formulate suitable research questions for further exploration within a multidisciplinary workshop.

Using the exemplar of Barrett’s oesophagus, the workshop was designed to evaluate the ethical and legal factors that might be generated by the use of a risk tool for risk stratification and predictive prevention for two specific contexts: symptomatic referral of patients who present with symptoms to their GP, and surveillance of those with a known diagnosis of Barrett’s oesophagus. It also included consideration of more speculative uses. A briefing note was drafted for workshop participants, describing the epidemiology of Barrett’s oesophagus, the aims and phases of Project DELTA, the development of a novel risk-algorithm which could be applied to electronic health records to identify those at increased risk of Barrett’s oesophagus, and some potential ethical and legal factors that might be relevant.

Participants were recruited to this workshop on a purposive basis with the objective of ensuring representation from key stakeholder groups including software/algorithm/risk tool developers; ethical and legal experts; clinicians; patient representatives; collaborators from Project DELTA and representatives from professional organisations. The aims of the workshop can be found below in Table 1.

**Table 1:** Stakeholder workshop aims and objectives.

Workshop aims and objectives
• To explore how a risk tool for risk stratification and predictive prevention might be incorporated together with Cytosponge^TM^ in existing pathways for the early detection of Barrett’s oesophagus and oesophageal cancer; • To identify and evaluate ethical and legal factors that may be generated by the use of the risk tool in each of these contexts; • To consider how these factors may impact design and implementation of the risk tool; • To consider the implications of more speculative uses of the risk algorithm/risk tool, such as in asymptomatic patients, and the impacts of automating the process of data mining using artificial intelligence.

The multidisciplinary half-day workshop was held via a virtual platform in September 2022, attended by twenty one participants (including five researchers from PHG Foundation). After presentations describing the context for, and process of, developing the risk algorithm for oesophageal cancer, and for developing nurse-led clinics to administer Cytosponge^TM,^ the workshop took the form of semi-structured plenary discussion, divided into two sessions. The focus of the first session was on exploring the ethical and legal considerations around the implementation of the risk tool into existing pathways (e.g. for symptomatic referrals and Barrett’s surveillance.) The questions put to participants were the following:

1. Are there are any particularly pertinent legal and ethical considerations that stand out to you when thinking about the use of risk algorithms for symptomatic patients?
2. How do these differ when thinking about:
  - Deployment in person
  - Deployment without face-to-face contact
3. Turning to the surveillance pathway where patients already have a diagnosis of Barrett’s oesophagus, are the proposed ethical and legal implications of using the risk algorithm substantially different?

The second session focused on future more speculative uses of the risk algorithm, the potential for the involvement of AI for data mining, and the implications of these developments. Participants were asked to reflect upon the following scenarios:

1. Screening asymptomatic individuals through batch processing risk stratification. This could take the form of:
  - Stratification within primary care using GP records
  - Stratification using centralised records as part of a wider screening programme, for example under the auspices of the National Screening Committee
2. The possible direct-to consumer use of the risk tool, through an app or webpage, without oversight from a healthcare professional
3. The potential use of AI in the future (either fully or partially) for data mining, and whether this raises any new considerations

Written informal consent to take part in the workshop under Chatham House Rules was obtained from each participant via email in response to their invitation. Participants also provided verbal consent to participate at the start of the workshop, where they were reminded of the terms of reference for participation, including that all contributions would be de-identified, and that the outputs from the workshop would include an academic paper and workshop report. A recording of the workshop was taken to supplement the notes drafted by PHG Foundation researchers during the workshop. A more detailed report of this workshop is available in S1 File.

The notes from the workshop were analysed by two researchers (TB and AH), who selected and organised content in accordance with key themes. Text fragments were extracted from the notes and iteratively coded to capture the key opportunities and challenges raised by the use of risk prediction algorithms for oesophageal cancer. These were then analysed against the results from the literature searches, and areas of convergence/divergence noted. Two researchers performed each step, with discrepancies resolved through discussion.

## 3. Results

As a result of this analysis we have identified a range of ethical, legal and associated practical challenges that arise in the implementation of risk algorithms for early detection and screening of oesophageal cancer in primary care. We have grouped these challenges under five related headings: supporting clinicians to use cancer risk prediction algorithms; bias, equity and fairness; ensuring transparency and appropriate risk communication; managing resources; and avoiding exceptionalism. We discuss the implications of these results, taking account of developments that may arise in stratification and screening, such as the adoption of AI driven technology in risk prediction, expansion of target screening and risk assessment beyond primary care, and the inclusion of genomic risk factors, in the subsequent Discussion section.

### 3.1 Supporting clinicians to use cancer risk prediction algorithms

A primary theme of discussion in our workshop and arising through our scrutiny of relevant literature, concerns the need to inform and train clinicians to adopt and use cancer risk prediction algorithms, so that they are supportive of the adoption of the algorithm into practice, and confident in using it.

"*We talk a lot about patients buying into risk stratification tools and understanding it*, *but I think clinicians need to buy into it as well*. *Clinicians need to understand the mechanisms behind why we have made these decisions and what is factored into it*.” (Clinician)

In this regard, ethical imperatives and practical issues are heavily entwined and it is clear that GP preparedness to use a risk prediction algorithm is a key component of effective implementation for early detection of cancer. Studies report mixed results in terms of clinician support for the use of cancer risk algorithms. A recent review of risk stratification of population-based cancer screening found that, in principle, it was acceptable to the majority of healthcare professionals [30]. However, evidence about the use of cancer risk calculators is more mixed. One study found moderately high awareness and a generally positive attitude towards cancer risk calculators amongst participants at the start of the study [20]. Another, exploring implementation of cancer risk tools, showed that the majority of GPs found the RATs useful in consultations and their use heightened GPs’ awareness of potential cancer symptoms, reminded and alerted them to potential cancer risks and affected their referral thresholds [31]. On the other hand, there is also evidence of reluctance amongst GPs to use cancer risk algorithms. Reasons for this reluctance include difficulty introducing the tool into the workflow of the consultation, or fear of alarming their patients if risk information is presented too explicitly [32]. Studies also found that clinical experience and belief in clinical intuition were determinants of tool use [32]. Many of these barriers are likely to apply equally to the use of CanPredict. Adequate training, evidence of clinical utility, and reassurances around liability could support clinicians to use the risk tool appropriately.

#### 3.1.1 Training and understanding

Workshop participants indicated that gaining support will, to some extent, depend upon ensuring that clinicians have a sufficient level of understanding around the development of the algorithm, and how it should be deployed in clinical practice.

“*To be able to explain where the algorithm came from*, *how it was derived (from what data and populations)*, *what the main inputs are*, *and what the relative risks are of those inputs I think fulfils the legal obligations of GDPR explicability*, *but also*, *I hope*, *gives clinicians and interested patients enough information to be able make up their own minds about the quality of the data and evidence and how much weight they should put on it*.*”* (Humanities researcher)

Explainability and interpretability should be prioritised for two purposes: first, this transparency helps to foster trust in the algorithm amongst clinicians, and second, it equips them to have confident and meaningful interactions with patients about their results. Studies have shown that sharing the risk tool during consultation aided communication, helped GPs explain to their patients why they would be referred, or not [31] and helped reassure anxious patients and justify legitimate concerns [33]. However, it is unclear what degree of understanding is sufficient to realise these benefits, particularly around how the risk estimates are derived. Too much information could be overwhelming, but too little may hinder trust, understanding and therefore adoption. A study investigating whether GPs change their referral decisions in response to an unnamed algorithm found that providing information about the algorithm (such as its derivation, validation and accuracy) does not have a discernible effect on decisions, but results in a more positive GP disposition towards the algorithm [20]. Supportive activities (such as training) that accompany the implementation process of the cancer risk tool are also key to GPs’ acceptance and perhaps more crucially, accurate reading of the tools [31].

#### 3.1.2 Demonstrating clinical utility

Lack of proven clinical utility of risk prediction models may also act as a barrier to uptake. The data on predictive utility (discrimination and calibration) of risk prediction models for cancer suggest that some have potential for clinical application. However, the focus on implementation and impact is much more recent and there remains considerable uncertainty about their clinical utility and how to implement them in order to maximise benefits and minimise harms such as over-medicalisation, anxiety and false reassurance [34]. Therefore, establishing effectiveness of the tool may be an important facilitator of GP uptake, although not sufficient on its own [31]. In a study exploring service users’ (adults without a cancer) and primary care practitioners’ perspectives on the barriers and facilitators to implementing a cancer risk assessment tool, some service users felt that the use of the QCancer® tool in patient consultations should be further evaluated for effectiveness on rates of referral, investigation, diagnosis or overdiagnosis, to enable a full comparison with current practice [35].

#### 3.1.3 Aiding cancer referral decision-making

One aim of incorporating an algorithm into a disease pathway is to optimise referrals for interventions such as Cytosponge^TM^ testing and endoscopies. However, whilst the use of a risk prediction tool might improve the consistency of clinical decision-making, it does not guarantee a change in clinician’s referral behaviours. Qualitative research indicates that GPs take a cautionary approach to risk results generated by an algorithm and might be more likely to adopt a risk estimate if it generates an increased risk rather than a decreased risk. In a study where 157 GPs responded to a series of clinical vignettes online so that investigators could see whether GPs change their referral decisions in response to an unnamed algorithm, GP’s were reluctant to withhold referral if the algorithm generated a low risk (e.g. less than the NICE guidance cut-off of 3%) [20]. It was found that they were more likely to change a referral decision if they had initially underestimated rather than overestimated risk. There may be circumstances where the clinical judgement of the health professional, and the recommendation from the risk tool for a particular patient, are not aligned, however the risk tool is intended to support (rather than override) clinical decision-making [35]. This was also emphasised by participants in the workshop, who commented that

“*There is a distinction between [the algorithm acting as] additional information*… *and making a decision just based on an algorithm*.*”* (Researcher)

They added that GPs have the capacity to refer outside of the NICE NG12 suspected cancer referral guidance and that the same flexibility would apply to any recommendations made by the algorithm.

“*GPs have the capacity to refer outside the NG12 and the algorithm-worked-out referral criteria*, *and GPs will always have the ability to refer any patient that they are worried about regardless of algorithms and other guidelines”* (Clinician*)*

Other qualitative cohort studies reinforce that these tools should not supersede clinical judgement and other guidance; rather they are an adjunct to the diagnostic process [33, 36]. A review of the role of GP’s ‘gut feelings’ in primary care suggests that some GP’s act on gut feelings (conceptualised as a rapid summing up of multiple verbal and non-verbal cues in the context of their clinical knowledge and experience) in making referral decisions but they may be associated with intuitions about patients being generally unwell rather than being at risk of cancer. These intuitions are often excluded from referral guidance, and are therefore poorly recorded and evidenced [37]. However some participants felt that more weight should be given to these feelings if there was sufficient justification.

*"We can have guidance and guidelines*. *I think there is a danger where they become so rigid that they become restrictions on the ability to deliver individually tailored care where there are risk factors and presentations*, *or indeed patient concerns*, *which are understandable and legitimate*, *and give rise to a reason to deviate from guidance*.*”* (Clinician)

In the context of oesophageal cancer, more work is needed to determine how, and to what degree, estimates from tools like CanPredict differ from GP’s intuitive estimates made without the support of the algorithm. It may be that GPs demonstrate a high degree of decision appropriateness without the use of an algorithm, although more research is needed to compare decision appropriateness pre and post algorithm, and to demonstrate the impact that CanPredict will have on referrals.

#### 3.1.4 Providing greater clarity around liability

Clinicians in the workshop expressed concern about generating an increased volume of risk information that may require intervention, which would inevitably lead to an increase in the number of referrals and more work for already overstretched primary care professionals. Studies have identified this as a barrier to the use of cancer decision tools more generally.

Although intended to support clinicians in their decision making, there was also concern around the extent to which clinicians could in future be obligated to act on the results of the risk tool, and that apprehension around liability may pose an additional barrier to clinical uptake. Participants spoke of “*the risk of not following up a risk*” (Clinician) and the possible disincentive this could pose to the adoption of the risk algorithm. Participants reflected on the fact that if clinicians are not informed of a patient’s risk, then they do not have to act on it, which may be perceived as a reason against implementation.

“*The potential liability which accrues to a physician or organisation which has the information about increased risk but doesn’t act upon it*, *creating a potential future liability under tort for negligence*, *and where that might sit*, *is important where we have an already overstretched primary care system*.*”* (Clinician)

Uptake could be impeded if clinicians felt that there was a medicolegal threat to their decision from patients who were later diagnosed with cancer after a cancer risk tool had highlighted an increased risk, but a decision had been made not to investigate or refer [33, 38]. Therefore, clear guidance and reassurance about how to manage conflicts between the risk tool and professional judgement may be necessary for successful implementation.

#### 3.1.5 Supporting clinician’s work processes

Although there are challenges to overcome, the use of a risk tool might have significant benefits beyond more accurate stratification of patients. It is possible that CanPredict will improve consistency in clinical decision-making, assist with vaguer presentations, and may even have an educational effect [38]. One study found that the use of cancer risk assessment tools gradually improved primary care clinicians’ decision-making over time [20] and another finding that they alerted GPs to the potential significance of more opaque signs and symptoms of cancer [31]. Risk calculators may have a role as training tools, enabling GPs to internalise the weighting of risk factors. Therefore the evidence conducted on these tools to date suggests that incorporating CanPredict into primary care clinical systems could prove valuable provided that it is designed and implemented in a way that supports clinicians’ work processes, and that awareness and education surrounding the tool is prioritised.

### 3.2 Bias, equity and fairness

As with all algorithms, it is crucial that the training dataset is representative of the population to which it will be applied, in order to promote equitable care. If the data used to train the algorithm are more representative of a subset of this population, the predictions from the model may also be systematically worse for underrepresented groups. This was emphasised by workshop participants.

“*With the modelling process you have to be aware of the limitations of the data*, *its coverage and whether it is representative”* (Researcher)

However, they highlighted that the QResearch dataset is comprehensive (as over 99% of the general population are registered with a GP). It also links with hospital episode statistics and cancer registration data so will capture the 22% of patients with oesophageal cancer that present in emergency care, rather than through primary care referrals [39]. Opportunities for early detection of risk may be reduced in the subset of individuals who self-medicate with over-the-counter proton pump inhibitors (PPIs) as in some instances there would be no record of medication use on their EHR.

Some of the challenges around equitable access arise as a result of *how* the risk tool is implemented, and whether it could have a knock-on effect on social inequalities. For example, one participant expressed concern that if the risk tool is used during GP appointments initiated by symptomatic patients (rather than applied to all EHR records held by the GP practice to identify those at high risk) then individuals who have not sought guidance or medication from their GP will potentially be disadvantaged by being ineligible for targeted screening [40].

“*There is a difference where the algorithm is being applied without talking to people [i*.*e*. *batch processing at a GP practice level*, *as opposed to symptomatic patients presenting in primary care]… that may in a way be more equal*.. *but if you have a conversation about whether someone’s happy to have an algorithm make a decision about them and their care*, *if they are not happy then what happens*? *What happens to people who don’t want that risk assessment*?*”* (Humanities researcher)

Participants also noted that there are inequalities in how people access treatments, and if the algorithm is trained on data solely derived from patients who have accessed treatment then that bias could be replicated in future applications of the algorithm. Prioritising access for the 20% of the population in the lowest deprivation quintile will ensure that efforts are aligned with NHS England’s Core20plus5 approach to reduce healthcare inequalities [41], for which earlier diagnosis of cancer is one of the clinical areas of focus.

Perceptions of fairness and bias were raised as being a critical component for building patient support:

*“A big concern for public acceptability are notions of perceived fairness*. *Why is she getting the test and not me*?.. *Why am I not going straight to endoscopy*?*… Explainability is also important for resolving those concerns*, *particularly to the extent that categories used for the risk prediction tool relate to socially salient categories around sex*, *race*, *ethnicity*, *or anything where there is a perceived*, *and perhaps real*, *issue of fairness*… *Everyone might understand what the risks involved are but may still feel uncomfortable about the notion that they are being excluded or other people are being excluded on these bases*.*”* (Humanities researcher)

It was suggested that this perceived lack of fairness could be resolved through communication with the patient.

*“When you explain to people about the test*, *you might want to explain “Is it good for you to have the test or not*?*” Another question is “Why are you getting the test*, *not her*?*” Those are two different questions*.*”* (Humanities researcher)

Workshop participants highlighted that when stratifying the population to receive interventions where resources are finite, prioritising some individuals for an intervention inevitably leads to the deprioritisation of others for that intervention. Transparency around the risk factors used in a stratification process could help reassure people that this is done ‘fairly’ and build trustworthiness in health systems more generally.

### 3.3 Transparency and risk communication

The format in which risk information is presented is a key aspect of implementing a risk algorithm/tool, as it affects both clinicians’ use of the tool and patients’ understanding and perception of risk, which sits at the heart of any discussion about informed decision-making.

It is not clear, however, how to present this risk information to patients and literature on this topic, emphasising that there is no single ‘correct’ approach to risk communication. We know from the field of cardiovascular disease that an individual’s perception of their risk does not always reflect their population level risk, and that patients may struggle to understand absolute and relative risks [42] and this challenge was reinforced by workshop participants.

*“It is a real challenge*, *very often*, *for people to understand the background risk*, *the relative increased risk and the absolute increased risk when it comes to making an informed decision…So there are challenges about the public understanding of risk*.. *which has to sit at the heart of any informed decision-making with individual patients based on a risk algorithm”* (Clinician)

*“When you’re explaining risk to people*, *it’s about what happens to a group of patients with similar characteristics*, *which is different to saying “this is what’s going to happen to you”*. *That framing and how it’s portrayed is crucially important*.*”* (Researcher)

However, they remarked that there is a strong body of evidence to draw on regarding best practice for explaining risk results and the uncertainties around them. This suggests that in order to promote understanding, using a combination of strategies for communicating risk may be most effective, including using descriptive terms (low, medium, high etc.), numerical data and visual aids/graphics, enabling a more tailored approach. Additionally, a qualitative study exploring the perspectives of service users and primary care practitioners found that communication of cancer risk information could be enhanced in the following ways: personalising risk information; involving patients when using the tools; being open and honest; and providing time for listening, informing, explaining and reassuring patients in a professional manner [43].

Participants felt that it is important to be able to explain not only the patient’s result but also how it was calculated and why a recommendation has been made. Participants suggested that one example of this is the PREDICT online tool for breast cancer, that helps patients and clinicians see how different treatments for early invasive breast cancer might improve survival rates after surgery [44]. It has different layers of explanation so that information provision can be tailored to the preferences of the patient. On the webpage, patients are able to see what the risk factors are, and should they wish to, the relative risks of each of those factors and even the actual algorithm. This satisfies the legal requirement to provide information under the GDPR in ways that facilitate patient understanding, thus meeting additional legal and ethical obligations for transparency.

In discussion, participants raised the challenge that effective risk communication is more achievable (although remains challenging) in instances where symptomatic patients present to primary care, and benefit from face-to-face risk communication in an environment where a clinician can explain the reasons behind a risk result. An alternative scenario, however, may be that the algorithm is applied to the GP practice database to identify high risk patients, who are subsequently contacted by letter, inviting them to have a Cytosponge^TM^ test. One workshop participant highlighted the difference between these scenarios.

*“If you have sat down with your doctor… you are in a decision-making environment and the doctor can explain ‘these are the inputs*, *this is why we are considering them and this is why you are at higher (or lower) risk and these are the options that are available to you now*.*’ That is a very different thing from suddenly being referred on the basis of an algorithm that you don’t know is being applied… These are two different scenarios in terms of the amount of information that you as a patient are having about what is happening to you and they have different ethical and legal implications*.*”* (Humanities researcher)

Additional challenges are raised where the healthcare system initiates contact with the patient rather than vice versa. This potentially changes the nature of the activity from a consultation initiated by a patient about the symptoms they are experiencing, to the unsolicited screening of a ‘healthy’ patient. This is significant because the balance of risks and harms might be different where a patient actively seeks advice compared to a screening context, where the patient believes themselves to be healthy. At the point of receiving a letter, an accompanying information leaflet should explain why the patient is being contacted, the implications of testing/declining testing and acknowledge any uncertainties that may arise. Experience during the COVID-19 pandemic created a precedent for contacting at-risk adults about their future risk of disease. At-risk adults were informed that they were at highest risk of catching the disease and of suffering serious complications or dying from COVID-19, but not that the means of identification was through a risk algorithm [45].

Lastly, one participant raised the challenge that the act of inviting an individual signals that this is of benefit to them, undermining the expectation that they can and will act autonomously. There is a question around the extent to which language of the letter/ information leaflet can be used to mitigate against this, and promote patient choice with the aim of information provision rather than persuasion. After all, the fundamental purpose of risk communication is to help patients understand the risks and benefits of the options they face so they can make informed choices between them. That said, it is not unusual to invite individuals for screening on the basis of risk factors, and learnings relating to information provision, language and framing can be drawn from existing screening and early detection programmes.

### 3.4 Managing resources

The introduction of a risk tool like CanPredict could have implications for clinicians’ workload, both in primary and secondary care. It has potential as a targeted screening tool to triage symptomatic patients who are at higher risk of oesophageal cancer for further investigation, potentially using Cytosponge^TM^. Although oesophageal cancer is rare, the numbers of people at risk of Barrett’s oesophagus are considerable in Western countries, since 10–15% of the adult population are affected by gastroesophageal reflux disease [46]. Participants commented that targeted screening of this group represents a considerable implementation challenge raising issues of scalability, and queried the impact that this will have on an already overstretched health system. Delivering Cytosponge^TM^ at scale will require more healthcare professionals to perform tests (as they cannot be self-administered), infrastructure to conduct analysis on the samples, and may lead to more patients making contact with primary care. However, low-cost, minimally invasive tests are not available for some of the rarer cancers, suggesting that population wide approaches for early detection of Barrett’s oesophagus as a precursor to oesophageal cancer may be more feasible than for other cancers.

Despite these concerns about the need for resources to support a new service or screening programme, workshop participants also raised the opportunity costs of failing to implement risk assessments and screening where the evidence indicates that it will lead to population level benefit.

*“If we have good data to support a screening programme and choose not to implement it*, *that also raises ethical considerations…we need to think about the ethical dimension of not implementing something that we think is of benefit at a population level where it is cost effective within the existing screening scheme*.*”* (Clinician)

It is possible that some GPs may also choose to use a predictive risk algorithm to supplement their decision making when symptomatic patients seek advice from them on an opportunistic basis. Evidence suggests that GPs may encounter a tension between limiting the numbers of referrals for further investigation for economic reasons, whilst simultaneously wishing to facilitate the earlier detection of cancer in at-risk patients.

Although GPs have been shown to make appropriate cancer referral decisions [20], it is likely that the use of an oesophageal cancer risk tool for risk stratification will re-categorise some patients. Challenges arise in navigating instances where an individual’s risk management recommendation changes as a result of the use of a risk tool, particularly where it leads to a reduction in services/interventions rather than additional ones. However, this challenge may be mitigated if GPs are likely to err on the side of caution and refer in line with their own assessment, even if the risk tool indicates that referral is not required.

### 3.5 Avoiding exceptionalism

Workshop participants cautioned against any possible exceptionalism around the use of a risk tool in this context.

*”We probably shouldn*’*t get hung up on the fact that it’s an algorithm*. *We use algorithms all the time.” (Researcher)*

Risk tools to predict the current or future risk of disease are used as part of a variety of risk assessments in primary care (albeit inconsistently), and NICE guidelines have recommended the use of QRISK2 for cardiovascular disease risk assessments since 2015 [47]. This tool is sufficiently similar to others, that those involved in its development and implementation have opportunities to learn from the implementation of other algorithms in order to embed it into routine practice.

*“If we were thinking about a situation where you were running an algorithm on GP records and then inviting people who hit those criteria to come in and have a Cytosponge, that is very similar to existing screening programmes where the leaflet that they get explains that they are being invited because we offer screening on the basis of gender and age*… *I don’t know whether it is overcomplicating it to try and think of it as a totally different thing if you’re just explaining to people that they are being invited on the basis of their sex, age, prescription history and whatever else is going into that model… The fact that the algorithm is transparent and not a black box means that it ought to be possible to explain to people in much the same way as we do with existing programmes. (Humanities researcher)*

It was also questioned whether the use of an algorithm, in the context of early detection of oesophageal cancer specifically, is a significant departure from existing practice.

*“We have been using risk stratification techniques in our guidelines in secondary care for Barrett’s surveillance for quite a long time… The use of an algorithm isn’t really new”* (Clinician)

In secondary care, risk stratification techniques are already used for Barrett’s oesophagus surveillance and the introduction of a risk tool applies a number to known risk factors and automates a process that already takes place. Quantifying risk with an algorithm/risk tool could even enhance the consistency of clinical decision-making, and although the use of a risk algorithm to stratify patients into risk groups is not novel, the Cytosponge^TM^ sampling method is, providing a level of actionability that has not previously been feasible.

## 4. Discussion and future considerations

The results of our research suggest that there are multiple ethical and legal/regulatory considerations that should be taken into account when considering how targeted symptomatic screening for Barrett’s oesophagus incorporating risk prediction algorithms should be implemented. Many of these are generalisable to other settings and contexts, such as requirements for fairness, transparency, and equity. However, there are some specific challenges that need to be addressed when stratifying the primary care patient population to identify those at most risk of Barrett’s oesophagus using risk prediction algorithms. First, by relying on electronic health records from primary care data, stratification using those records potentially replicates gaps in coverage and potential biases in that data. For example, certain groups (non-white ancestries, those with no fixed address) may find it more difficult to access primary care services although professional guidance states that anybody, regardless of nationality and residential status, may register and consult with a GP without charge [48].

Second, since the risk algorithm is a stratification tool, typically applied to the at-risk population as a whole, it may be less sensitive when applied to certain groups if there are small numbers involved, such as young people and rarer ancestral groups. Ethnicity was included as a final predictor variable in Hippisley-Cox’s final model for oesophageal cancer (defined in terms of a choice from nine different categories). They found that white men had a higher risk than any other recorded ethnic group, and for women, Bangladeshi ancestry had a higher risk [5]. Targeting these groups could help to resolve some of these uncertainties. However, another way of mitigating these challenges might be to utilise artificial intelligence/machine learning (AI/ML) to incorporate a greater range of risk factors for Barrett’s oesophagus, and enable more nuanced pattern recognition, potentially resulting in greater diagnostic yield and improved detection and disease prevention.

The adoption of AI methods to bolster risk assessment is one of potential developments that foreseeably could have an impact on early detection and screening. In the rest of this section, we consider the extent to which these methods might generate additional ethical, legal or practical challenges which will need to be addressed.

### 4.1 Utilising artificial intelligence to bolster risk assessment

Artificial intelligence/machine learning (AI/ML) covers a multitude of different technologies across many different applications. There are many claims made about how potentially transformative AI/ML could be. But a lack of consensus about the nature of AI/ML given that many technologies follow existing statistical methods, and the context of health where predictors of disease are often imperfectly and partly measured and many of the causal mechanisms are unknown, makes these claims difficult to assess.

In theory, AI/ML could provide a means of improving the accuracy of individual risk assessments for oesophageal cancer, while safeguarding specificity and sensitivity. In a research setting, Briggs et al developed five probabilistic machine learning classifiers each utilising basic demographic variables, symptoms and laboratory test results with the aim of maximising recall while minimising the proportion of unnecessary referrals. Compared to a current UK oesophageal-gastric cancer risk-assessment tool (ogRAT), the authors claim that machine learning identified 11% more cancer patients with negligible effect on false positives or up to 25% more patients with a slight increase in false positives [49].

Moving beyond existing statistical methods, using black-box algorithms has potential to streamline the risk assessment process and increase throughput, and even improve the consistency of decision-making over physician only processes, but potentially raises a number of additional challenges. It will be important for health professionals using these automated risk tools for their patients to understand both the internal logic of the algorithm (what drives the machine learning to make the decision it did for this particular patient), and the evidence on which any decision was based (transparency about the nature of the risk factors and protective factors selected for a particular outcome, and information about how they are combined and weighted for individual risk prediction). Since black box algorithms are, by definition, dynamic and opaque, using artificial intelligence for risk assessment without sufficient transparency and explanation could substantially undermine the trust of health professional users and their patients. In the short term, one way of mitigating these potential shortcomings is to ensure that there is the potential for human oversight of any decision made [50]. This will help to ensure continued trust and confidence in these algorithms as they are introduced more widely into practice. This will also help to mitigate the potential impact of artificial intelligence applications being classified, for data protection purposes, as automated profiling [51] which requires specific safeguards to be in place, such as the data processing to be authorised by consent, or for restrictions to be placed on when such processing is lawful, such as when it is necessary for the performance of a contract between the patient (the data subject) and the health provider (the data controller) [52].

The potential for wholly automated risk assessment and referral processes in future, highlights the urgent need to determine the nature, volume and timing of information about the risk tool provided to health professionals, and potentially to patients. If the ubiquity of risk assessment within current medical practice suggests that it should be regarded as a necessary part of clinical judgement, what impact might the automation of patient pathways have on what patients and publics are told about the activity of risk profiling?

Although these are ethical questions about future professional practice, they also have regulatory ramifications, as regulatory agencies, such as the UK’s Medicines and Healthcare products Regulatory Agency (MHRA) grapple with the evidence, performance standards, and surveillance that should apply to software being used for risk assessment [53]. Since the developer’s intended purpose of any risk tool is critical in framing the degree of regulatory oversight required, key questions about the future users of risk tools, (e.g. whether these are ‘expert’ health professionals, or ‘lay’ patients/publics) and the reliance that they place on the tool as part of any decision making process (i.e. whether it is used to ‘guide’ or to ‘drive’ decision making) will likely be critical to future implementation policies.

### 4.2 Expanding targeted screening and risk assessment beyond primary care

The main focus of our research is on ‘targeted screening’ of patients registered with a GP. Arguably however, developing a risk tool that could utilise additional sources of data, such as supermarket or pharmacist sales of antacids associated with loyalty card information, together with self-reported symptoms of gastro-oesophageal reflux disease (GORD) which were not captured in electronic primary care data, could have the potential to identify at risk individuals who are not well represented in GP records. Proof of principle research adopting this approach has reported higher rates of detection of ovarian cancer linked to non-steroidal anti-inflammatory use [54], and it is feasible that similar methods could be used for early detection of Barrett’s oesophagus provided that retailers and consumers are willing for their data to be shared in this way.

For example, as part of Project DELTA a mobile screening site administering Cytosponge^TM^ was set up at three locations characterised by different levels of deprivation and population density. At each site, it was possible for patients to refer themselves for Cytosponge^TM^ provided they met certain criteria including symptoms of dyspepsia and medication use. Although numbers of self-referred patients were relatively small at each site, a consistent proportion of those went on to be eligible for follow-up by Cytosponge^TM^ or endoscopy. This suggests that it might be theoretically possible to devise a pathway for patients who could be at higher risk of oesophageal cancer, involving screening using a non-invasive diagnostic test such as Cytosponge^TM^, without prior referral from a GP.

However, there are a number of potential ethical, legal and practical barriers to the pathway being extended to a lay user. For example, CanPredict is currently intended for use by health professionals working in primary care as a clinical support tool. If it is intended for medical use, the predictive risk algorithms underpinning the risk tool, and the risk tool itself, could qualify as a medical device either under European Medical Devices Regulations (as a transitional measure) or UK Medical Devices Regulations [55]. If this is the case, the software will have to be UKCA or CE marked, be safe and effective, and meet requirements for transparency, governance and quality assurance [56]. As discussed above, it will be more likely to be classified as a device if the calculations involved are not easily verified by the health professional and/or it drives clinical action rather than informing it.

Making the risk tool publicly accessible as a web based calculator which can be accessed by patients without professional oversight, potentially increases the possibility of it being relied upon to determine future treatment and management by non-expert users. This potentially changes the regulatory status of the predictive risk algorithm from a low risk (Class I) to a medium risk (Class IIa) medical device, and requires more explanation and transparency about how the risk predictor should be used, and the potential harms and benefits involved. The current regulatory landscape for medical devices in the UK is highly dynamic and complex while regulators and developers adapt to the changed regulatory landscape following Brexit. While the MHRA has a road map for future reform of relevant regulations which prioritises developing a proportionate and effective regulatory regime which facilitates innovation while ensuring safety and effectiveness [53], the details of post-Brexit software regulation in the UK remain unclear. The uncertain and complex regulatory environment for risk prediction algorithm development in the UK is a significant barrier to implementation [57].

Irrespective of these regulatory considerations, in the short to medium term, it seems likely that health professionals will need to be involved in referring patients for downstream investigations (endoscopy and/or Cytosponge^TM^) for several reasons. Their role will include ensuring that the harms associated with those tests are proportionate to their potential benefits, and in authorising downstream tests if they are provided by the NHS. Potential downstream costs include sample collection from the oesophagus using Cytosponge^TM^ or endoscopy, analysing that sample for evidence of cellular changes indicative of disease, reporting results to health professionals and their patients, and treatment of any affected areas of the oesophagus, through endoscopic ablation or in the most severe cases, surgery. Understanding the economic costs relating to these different elements of the patient pathway will be key in building trust and influencing uptake, and work to generate evidence of cost-effectiveness within Project DELTA is currently underway [58].

In their recent paper Hippisley-Cox et al envisage that risk prediction algorithms could be integrated into national clinical computer systems to identify high risk patients, for prevention, diagnostic triage and targeted screening provided that such pathways can be shown to be cost-effective [5]. Although cost-effectiveness is important, the most decisive factor in driving implementation of the novel pathway will be evidence of clear clinical validity and utility on rates of oesophageal cancer. A forthcoming research trial (BEST4) is in progress, which involves a large randomised trial to evaluate the effectiveness of an offer of Cytosponge^TM^ to patients on medication for heartburn symptoms and whether this could improve oesophageal adendocarcinoma- associated mortality, thus demonstrating clinical validity and utility for early detection of Barrett’s oesophagus [59]. It is hoped that results from this study will underpin the implementation of a publicly funded screening programme in which those found to be at highest risk are referred for Cytosponge^TM^ testing. Determining eligibility for such screening programmes will be critical. Extending eligibility beyond primary care might mitigate concerns about targeted screening being potentially inequitable, but may have the potential to increase the numbers of false positives and negatives from the pathway, reducing test sensitivity and specificity.

Finally, the future provision of a direct-to-consumer service which provides a package of risk stratification, sampling (where patients self-administer Cytosponge^TM^) and remote analysis and reporting is feasible. However, this would seem unlikely in the near-term as it would result in increasing pressure on scarce NHS resources for timely and effective endoscopy and treatment for the minority who screen positive and require confirmation of a positive diagnosis and treatment.

### 4.3 Refining predictive risk algorithms to include genomic risk factors

Another development which may significantly impact cancer risk prediction is the inclusion of genomic factors as part of risk assessment. At present, the risk prediction algorithms which have been developed for use in primary care do not include genetic or genomic markers associated with oesophageal cancer because these are not yet routinely tested for or recorded in primary care [60–62]. However, understanding the evolution of oesophageal tumours over time, and being able to integrate successive genomic, histological and clinical records may drive the development of risk prediction algorithms for oesophageal cancer that incorporate genetic and genomic risk factors [12].

Non-inherited somatic, acquired variants such as p53 have a role in cancer development and have been implicated in most cases of Barrett’s oesophagus that progress. Immunohistochemical staining for p53 is increasingly applied for evaluation of Barrett’s surveillance biopsies [12] and is routine in evaluation of Cytosponge^TM^ samples within Project DELTA [7].

More speculatively, there is increasing interest in how cumulative polygenic risks which are weakly associated with a disorder can be used for stratification. Combining information from thousands or millions of these small effects, which are present in at least 1% of the population, can create a polygenic score. Since polygenic scores are normally distributed in a population, identifying an individual’s score and where it lies along this normal distribution can inform risk stratification [63]. At present, integrating polygenic risk scores into existing QCancer risk prediction algorithms provides only modest improvements in risk prediction for those at highest risk of colorectal cancer, creating insufficient justification for the generation and use of polygenic risk scores in the general population [64]. Polygenic risk scores for oesophageal cancer are currently in an early stage of development and there is no evidence to suggest that they might be useful for stratification.

Even if they were theoretically useful, a significant barrier to implementation would be the lack of routine accessibility to individual genetic and genomic data within primary care. However, there are a number of research projects which are set to generate genomic information at scale for certain cohorts of patients. One such project is the forthcoming Genomics England Limited Newborn Genomes Project which aims to screen 100 thousand newborns for a predefined set of rare, childhood onset, actionable genetic diseases [65]. Another, the Our Future Health research programme, has ambitious targets for generating polygenic risk scores, and potentially some rare genetic variants for up to 5 million UK participants. Together with programmes to deliver whole genome sequencing across clinical care within the NHS [66] the routine incorporation of genetic and genomic data within primary care records seems foreseeable in the medium term, although this will require very significant investment in digital infrastructure, data governance and training. These trends could result in the evolution of risk prediction algorithms and tools for oesophageal cancer that have the potential to detect subsets of individuals who have significant genetic risk factors and/or who are more likely to develop aggressive forms of the disease. These could be supplemented by digital reports which integrate multiple data sources to provide more detailed diagnosis and prognosis [67].

Another potential use for polygenic scores, might be to identify those at lowest risk of developing cancer, to inform decisions for population wide screening including decisions about who should be excluded from screening, or those who should receive screening more infrequently [68]. As we have already seen, withholding interventions from those at lowest risk is not straightforward and may raise additional ethical challenges even if logistically feasible. Given that there is currently no data on polygenic risk scores in Barrett’s oesophagus, the use of polygenic risk scores for exclusion also seems unlikely in the short to medium term.

## 5. Conclusions

Risk prediction algorithms potentially play a key role in early detection and screening for cancer, in stratifying those patients who have most to gain from onward referral for further investigation. In the context of Barrett’s oesophagus and oesophageal cancer, the CanPredict algorithm and Cytosponge^TM^ have been developed as complementary elements of a novel patient pathway, which could be used to offer targeted screening at scale. Such approaches could lead to improvements in population health, but our work suggests that there are a number of ethical and legal challenges that will need to be addressed for successful implementation.

These include challenges that apply to the use of risk tools broadly, such as the need to identify and mitigate against possible sources of bias, as well as others that are particularly exacerbated in the context of oesophageal cancer, such as the impact that scaling the use of the risk tool might have on resources. Developing risk communication strategies that take account of the use of risk algorithms has been shown to be particularly important for effective implementation, as is gaining support from clinicians. A number of strategies may be necessary to facilitate this support and promote uptake amongst primary health practitioners, including prioritising awareness and education, sharing data demonstrating the predictive utility of the risk tool and providing clear guidance and reassurance about how conflicts between clinical support tools and professional judgement might be managed. Strategies to promote equitable access should be prioritised, and could result in the adoption of batch processing where all at-risk individuals are contacted by the health system, despite the additional challenges raised by implementing risk tools for asymptomatic individuals.

This is a dynamic area and if we look to the future, it is feasible that such risk tools will be enhanced through the incorporation of genomic information about inherited or acquired risk or by using artificial intelligence to bolster risk assessment. This could further improve the accuracy of the risk stratification, but raises additional legal and ethical considerations, including around the degree of reliance that may be placed on the risk tool.

## Supporting information

S1 File Workshop report on adopting a risk tool for stratification and predictive prevention of oesophageal cancer. (DOCX)

## References

[1] Office for Life Sciences, Department of Health and Social Care, Department for Science, Innovation and Technology, Department of Business Energy & Industrial Strategy, Lord Bethell of Romford. Genome UK: The future of healthcare [Internet]. 2020 [cited 2023 Mar 2]. Available from: ttps://www.gov.uk/government/publications/genome-uk-the-future-of-healthcare

[2] Hippisley-Cox J, Coupland C, Vinogradova Y, Robson J, Minhas R, Sheikh A, et al. Predicting cardiovascular risk in England and Wales: prospective derivation and validation of QRISK2. BMJ. 2008 Jun 28;336(7659):1475–82. doi: 10.1136/bmj.39609.449676.25 18573856 PMC2440904

[3] Hall AE, Chowdhury S, Hallowell N, Pashayan N, Dent T, Pharoah P, et al. Implementing risk-stratified screening for common cancers: a review of potential ethical, legal and social issues. J Public Health (Oxf). 2014 Jun;36(2):285–91. doi: 10.1093/pubmed/fdt078 23986542 PMC4041100

[4] Hull MA, Rees CJ, Sharp L, Koo S. A risk-stratified approach to colorectal cancer prevention and diagnosis. Nat Rev Gastroenterol Hepatol. 2020 Dec;17(12):773–80. doi: 10.1038/s41575-020-00368-3 33067592 PMC7562765

[5] Hippisley-Cox J, Mei W, Fitzgerald R, Coupland C. Development and validation of a novel risk prediction algorithm to estimate 10-year risk of oesophageal cancer in primary care: prospective cohort study and evaluation of performance against two other risk prediction models. *The Lancet Regional Health—Europe*. 2023;32:100700. doi: 10.1016/j.lanepe.2023.100700 37635924 PMC10450987

[6] Pilonis ND, Killcoyne S, Tan WK, O’Donovan M, Malhotra S, Tripathi M, et al. Use of a Cytosponge biomarker panel to prioritise endoscopic Barrett’s oesophagus surveillance: a cross-sectional study followed by a real-world prospective pilot. Lancet Oncol. 2022 Feb;23(2):270–8. doi: 10.1016/S1470-2045(21)00667-7 35030332 PMC8803607

[7] Gehrung M, Crispin-Ortuzar M, Berman AG, O’Donovan M, Fitzgerald RC, Markowetz F. Triage-driven diagnosis of Barrett’s esophagus for early detection of esophageal adenocarcinoma using deep learning. Nat Med. 2021 May;27(5):833–41. doi: 10.1038/s41591-021-01287-9 33859411

[8] Odze R. Histology of Barrett’s Metaplasia: Do Goblet Cells Matter? Dig Dis Sci. 2018;63:2042–51. doi: 10.1007/s10620-018-5151-z 29998421

[9] Eusebi LH, Cirota GG, Zagari RM, Ford AC. Global prevalence of Barrett’s oesophagus and oesophageal cancer in individuals with gastro-oesophageal reflux: a systematic review and meta-analysis. Gut. 2021 Mar;70(3):456–63. doi: 10.1136/gutjnl-2020-321365 32732370

[10] Fitzgerald RC, di Pietro M, Ragunath K, Ang Y, Kang JY, Watson P, et al. British Society of Gastroenterology guidelines on the diagnosis and management of Barrett’s oesophagus. Gut. 2014 Jan;63(1):7–42. doi: 10.1136/gutjnl-2013-305372 24165758

[11] Hvid-Jensen F, Pedersen L, Drewes AM, Sørensen HT, Funch-Jensen P. Incidence of adenocarcinoma among patients with Barrett’s esophagus. N Engl J Med. 2011 Oct 13;365(15):1375–83. doi: 10.1056/NEJMoa1103042 21995385

[12] Killcoyne S, Fitzgerald RC. Evolution and progression of Barrett’s oesophagus to oesophageal cancer. Nat Rev Cancer. 2021 Nov;21(11):731–41. doi: 10.1038/s41568-021-00400-x 34545238

[13] Office for National Statistics. Cancer survival in England: adult, adults diagnosed between 2013 and 2017 and following up to 2018. Table 1. One-year and five-year net survival (%) with 95% confidence intervals (CI), for adults (aged 15–99 years) diagnosed between 2013 and 2017: England, 29 common cancers, by age and sex. [Internet]. 2019 [cited 2023 Mar 2]. Available from: https://www.ons.gov.uk/peoplepopulationandcommunity/healthandsocialcare/conditionsanddiseases/datasets/cancersurvivalratescancersurvivalinenglandadultsdiagnosed

[14] Office for National Statistics. Cancer survival in England: adult, adults diagnosed between 2013 and 2017 and following up to 2018. Table 4. Predicted estimates of one-year, five-year and ten-year net survival (%) with 95% confidence intervals (CI), for adults (aged 15–99) that would be diagnosed in 2018: England, 29 common cancers, by age and sex. [Internet]. [cited 2023 Aug 9]. Available from: https://www.ons.gov.uk/peoplepopulationandcommunity/healthandsocialcare/conditionsanddiseases/datasets/cancersurvivalratescancersurvivalinenglandadultsdiagnosed

[15] Lynch C. Cancer Research UK. 2019 [cited 2023 Mar 2]. Measuring up: How does the UK compare internationally on cancer survival? Available from: https://news.cancerresearchuk.org/2019/09/11/measuring-up-how-does-the-uk-compare-internationally-on-cancer-survival/

[16] Arnold M, Rutherford MJ, Bardot A, Ferlay J, Andersson TML, Myklebust TÅ, et al. Progress in cancer survival, mortality, and incidence in seven high-income countries 1995–2014 (ICBP SURVMARK-2): a population-based study. Lancet Oncol. 2019 Nov;20(11):1493–505. doi: 10.1016/S1470-2045(19)30456-5 31521509 PMC6838671

[17] National Institute for Health and Care Excellence. NICE guideline [NG12]. Suspected cancer: recognition and referral. [Internet]. 2015 [cited 2023 Mar 2]. Available from: https://www.nice.org.uk/guidance/ng12/chapter/Recommendations-organised-by-site-of-cancer 32212590

[18] Sirota M, Kostopoulou O, Round T, Samaranayaka S. Prevalence and alternative explanations influence cancer diagnosis: An experimental study with physicians. Health Psychol. 2017 May;36(5):477–85. doi: 10.1037/hea0000461 28240921

[19] Price S, Spencer A, Medina-Lara A, Hamilton W. Availability and use of cancer decision-support tools: a cross-sectional survey of UK primary care. British Journal of General Practice. 2019 Jul;69(684):e437–43. doi: 10.3399/bjgp19X703745 31064743 PMC6592323

[20] Kostopoulou O, Arora K, Pálfi B. Using cancer risk algorithms to improve risk estimates and referral decisions. Communications medicine. 2022;2:2. doi: 10.1038/s43856-021-00069-1 35603307 PMC9053195

[21] Usher-Smith JA, Silarova B, Ward A, Youell J, Muir KR, Campbell J, et al. Incorporating cancer risk information into general practice: a qualitative study using focus groups with health professionals. British Journal of General Practice. 2017 Mar;67(656):e218–26. 10.3399/bjgp17X689401 PMC5325664 28193618

[22] Hippisley-Cox J. Incorporating cancer risk information into general practice: a qualitative study using focus groups with health professionals. British Journal of General Practice. 2017 Apr;67(657):158.2–158. 10.3399/bjgp17X690161 PMC5565829 28360053

[23] Department of Health & Social Care. Policy Paper. A plan for digital health and social care. [Internet]. 2023 Jun [cited 2023 Aug 9]. Available from: https://www.gov.uk/government/publications/a-plan-for-digital-health-and-social-care/a-plan-for-digital-health-and-social-care.

[24] Recommendation 11.8 recommends the use of QRISK2 risk assessment tool to assess CVD risk for the primary prevention of CVD in people up to and including the age of 84 years. See National Institute for Health and Care Excellence. Clinical Guideline CG181. Cardiovascular disease: risk assessment and reduction including lipid modification [Internet]. 2014 [cited 2023 Mar 15]. Available from: https://www.nice.org.uk/guidance/cg181

[25] Hippisley-Cox J, Coupland C, Brindle P. Development and validation of QRISK3 risk prediction algorithms to estimate future risk of cardiovascular disease: prospective cohort study. BMJ. 2017 May 23;j2099. doi: 10.1136/bmj.j2099 28536104 PMC5441081

[26] Hippisley-Cox J, Coupland CA, Mehta N, Keogh RH, Diaz-Ordaz K, Khunti K, et al. Risk prediction of covid-19 related death and hospital admission in adults after covid-19 vaccination: national prospective cohort study. BMJ. 2021 Sep 17;n2244. doi: 10.1136/bmj.n2244 34535466 PMC8446717

[27] NHS. About omeprazole [Internet]. 2021 [cited 2023 Mar 2]. Available from: https://www.nhs.uk/medicines/omeprazole/about-omeprazole/

[28] National Institute for Health and Care Excellence. Scenario: Dyspepsia- proven GORD [Internet]. 2023 [cited 2023 Aug 9]. Available from: https://cks.nice.org.uk/topics/dyspepsia-proven-gord/management/dyspepsia-proven-gord/

[29] National Institute for Health and Care Excellence [Internet]. 2023 [cited 2023 Aug 9]. British National Formulary (BNF). Available from: https://bnf.nice.org.uk

[30] Taylor LC, Law K, Hutchinson A, Dennison RA, Usher-Smith JA. Acceptability of risk stratification within population-based cancer screening from the perspective of healthcare professionals: A mixed methods systematic review and recommendations to support implementation. PLoS One. 2023 Feb 24;18(2):e0279201. doi: 10.1371/journal.pone.0279201 36827432 PMC9956883

[31] Green T, Martins T, Hamilton W, Rubin G, Elliott K, Macleod U. Exploring GPs’ experiences of using diagnostic tools for cancer: a qualitative study in primary care. Fam Pract. 2015 Feb 1;32(1):101–5. doi: 10.1093/fampra/cmu081 25448163

[32] Chiang PPC, Glance D, Walker J, Walter FM, Emery JD. Implementing a QCancer risk tool into general practice consultations: an exploratory study using simulated consultations with Australian general practitioners. Br J Cancer. 2015 Mar 31;112 Suppl 1(Suppl 1):S77–83. doi: 10.1038/bjc.2015.46 25734392 PMC4385980

[33] Bradley PT, Hall N, Maniatopoulos G, Neal RD, Paleri V, Wilkes S. Factors shaping the implementation and use of Clinical Cancer Decision Tools by GPs in primary care: a qualitative framework synthesis. BMJ Open. 2021 Feb 19;11(2):e043338. 10.1136/bmjopen-2020-043338 PMC7896585 33608402

[34] Usher-Smith J, Emery J, Hamilton W, Griffin SJ, Walter FM. Risk prediction tools for cancer in primary care. Br J Cancer. 2015 Dec 22;113(12):1645–50. doi: 10.1038/bjc.2015.409 26633558 PMC4701999

[35] Akanuwe JNA, Black S, Owen S, Siriwardena AN. Barriers and facilitators to implementing a cancer risk assessment tool (QCancer) in primary care: a qualitative study. Prim Health Care Res Dev. 2021 Oct 7;22:e51. doi: 10.1017/S1463423621000281 34615569 PMC8527274

[36] Hamilton W, Green T, Martins T, Elliott K, Rubin G, Macleod U. Evaluation of risk assessment tools for suspected cancer in general practice: a cohort study. Br J Gen Pract. 2013 Jan;63(606):e30–6. doi: 10.3399/bjgp13X660751 23336455 PMC3529290

[37] Smith CF, Drew S, Ziebland S, Nicholson BD. Understanding the role of GPs’ gut feelings in diagnosing cancer in primary care: a systematic review and meta-analysis of existing evidence. British Journal of General Practice. 2020 Sep;70(698):e612–21. doi: 10.3399/bjgp20X712301 32839162 PMC7449376

[38] Dikomitis L, Green T, Macleod U. Embedding electronic decision-support tools for suspected cancer in primary care: a qualitative study of GPs’ experiences. Prim Health Care Res Dev. 2015 Nov;16(6):548–55. doi: 10.1017/S1463423615000109 25731758

[39] Elliss-Brookes L, McPhail S, Ives A, Greenslade M, Shelton J, Hiom S, et al. Routes to diagnosis for cancer–determining the patient journey using multiple routine data sets. Br J Cancer. 2012 Oct 20;107(8):1220–6. doi: 10.1038/bjc.2012.408 22996611 PMC3494426

[40] There is evidence to show that some subsets of individuals have more barriers to access and are less likely to present in primary care. See Fisher R, Fraser C. Who Gets In? What does the 2020 GP patient survey tell us about access to general practice? [Internet]. [cited 2023 Mar 3]. Available from: https://www.health.org.uk/news-and-comment/charts-and-infographics/who-gets-in

[41] NHS England. Core20PLUS5 (adults)–an approach to reducing healthcare inequalities [Internet]. [cited 2023 Mar 15]. Available from: https://www.england.nhs.uk/about/equality/equality-hub/national-healthcare-inequalities-improvement-programme/core20plus5/

[42] Gigerenzer G, Gaissmaier W, Kurz-Milcke E, Schwartz LM, Woloshin S. Helping Doctors and Patients Make Sense of Health Statistics. Psychol Sci Public Interest. 2007 Nov;8(2):53–96. doi: 10.1111/j.1539-6053.2008.00033.x 26161749

[43] Akanuwe JNA, Black S, Owen S, Siriwardena AN. Communicating cancer risk in the primary care consultation when using a cancer risk assessment tool: Qualitative study with service users and practitioners. Health Expect. 2020 Apr;23(2):509–18. 10.1111/hex.13016 PMC7104630 31967704

[44] NHS. Predict Breast Cancer [Internet]. [cited 2023 Aug 2]. Available from: https://breast.predict.nhs.uk

[45] Public Health England. COVID-19 vaccination: consent form and letter for adults [Internet]. 2020 [cited 2023 Mar 3]. Available from: https://www.gov.uk/government/publications/covid-19-vaccination-consent-form-and-letter-for-adults

[46] El-Serag HB, Sweet S, Winchester CC, Dent J. Update on the epidemiology of gastro-oesophageal reflux disease: a systematic review. Gut. 2014 Jun;63(6):871–80. doi: 10.1136/gutjnl-2012-304269 23853213 PMC4046948

[47] National Institute for Health and Care Excellence. Cardiovascular risk assessment and lipid modification, Quality standard [QS100] [Internet]. 2015 [cited 2023 Mar 3]. Available from: https://www.nice.org.uk/guidance/qs100/chapter/Quality-statement-1-Full-formal-risk-assessment-using-QRISK2

[48] British Medical Association. Patient registration [Internet]. 2022 [cited 2023 Mar 3]. Available from: https://www.bma.org.uk/advice-and-support/gp-practices/managing-your-practice-list/patient-registration

[49] Briggs E, de Kamps M, Hamilton W, Johnson O, McInerney CD, Neal RD. Machine Learning for Risk Prediction of Oesophago-Gastric Cancer in Primary Care: Comparison with Existing Risk-Assessment Tools. Cancers (Basel). 2022 Oct 14;14(20):5023. doi: 10.3390/cancers14205023 36291807 PMC9600097

[50] Redrup Hill E, Mitchell C, Brigden T, Hall A. Ethical and legal considerations influencing human involvement in the implementation of artificial intelligence in a clinical pathway: A multi-stakeholder perspective. Front Digit Health. 2023 Mar 13;5. 10.3389/fdgth.2023.1139210 PMC10043985 36999168

[51] Information Commissioner’s Office. Guide to the UK General Data Protection Regulation (UK GDPR) [Internet]. [cited 2023 Mar 3]. Available from: https://ico.org.uk/for-organisations/guide-to-data-protection/guide-to-the-general-data-protection-regulation-gdpr/

[52] UK General Data Protection Regulation. Article 22 (1)-(4).

[53] Medicines & Healthcare products Regulatory Agency. Software and AI as a Medical Device Change Programme—Roadmap. 2022 Oct.

[54] Brewer HR, Hirst Y, Chadeau-Hyam M, Johnson E, Sundar S, Flanagan JM. Association Between Purchase of Over-the-Counter Medications and Ovarian Cancer Diagnosis in the Cancer Loyalty Card Study (CLOCS): Observational Case-Control Study. JMIR Public Health Surveill. 2023 Jan 26;9:e41762. doi: 10.2196/41762 36701184 PMC9912145

[55] The Medical Devices Regulations [Internet]. No.618 2002. Available from: https://www.legislation.gov.uk/uksi/2002/618/contents/made

[56] Medicines & Healthcare products Regulatory Agency. Guidance: Medical device stand-alone software including apps (including IVDMDs) v1.10 [Internet]. [cited 2023 Mar 3]. Available from: https://assets.publishing.service.gov.uk/government/uploads/system/uploads/attachment_data/file/1105233/Medical_device_stand-alone_software_including_apps.pdf

[57] Hall A, Gehrung M, McCord M, Moss A, Hippisley-Cox J, Fitzgerald R. PHG Foundation. 2023 [cited 2023 Aug 16]. DELTA Position Statement. Available from: https://www.phgfoundation.org/research/assessing-ai-for-early-detection-of-oesophageal-cancer

[58] Project DELTA. About Project DELTA: Health economic and implementation research [Internet]. [cited 2023 Feb 23]. Available from: https://www.deltaproject.org/copy-of-about-3

[59] National Institute for Health and Care Research. BEST4: A Platform Trial to determine whether Cytosponge-biomarker technology reduces mortality from oesophageal cancer [Internet]. [cited 2023 Mar 6]. Available from: https://www.fundingawards.nihr.ac.uk/award/NIHR135565

[60] Ghosh P, Campos VJ, Vo DT, Guccione C, Goheen-Holland V, Tindle C, et al. AI-assisted discovery of an ethnicity-influenced driver of cell transformation in esophageal and gastroesophageal junction adenocarcinomas. JCI Insight. 2022 Sep 22;7(18). doi: 10.1172/jci.insight.161334 36134663 PMC9675486

[61] Dong J, Buas MF, Gharahkhani P, Kendall BJ, Onstad L, Zhao S, et al. Determining Risk of Barrett’s Esophagus and Esophageal Adenocarcinoma Based on Epidemiologic Factors and Genetic Variants. Gastroenterology. 2018 Apr;154(5):1273–1281.e3. 29247777 10.1053/j.gastro.2017.12.003 PMC5880715

[62] Ferrer-Torres D, Nancarrow DJ, Steinberg H, Wang Z, Kuick R, Weh KM, et al. Constitutively Higher Level of GSTT2 in Esophageal Tissues From African Americans Protects Cells Against DNA Damage. Gastroenterology. 2019 Apr;156(5):1404–15. doi: 10.1053/j.gastro.2018.12.004 30578782 PMC6441633

[63] Brigden T, Sanderson S, Janus J, Babb de Villiers C, Moorthie S, Kroese M, et al. Implementing polygenic scores into NHS Health Checks [Internet]. 2021 [cited 2023 Aug 16]. Available from: https://www.phgfoundation.org/media/491/download/PRS%20Implementation%20Report%2017%20Sept%202021.pdf?v=3&inline=1

[64] Briggs SEW, Law P, East JE, Wordsworth S, Dunlop M, Houlston R, et al. Integrating genome-wide polygenic risk scores and non-genetic risk to predict colorectal cancer diagnosis using UK Biobank data: population based cohort study. BMJ. 2022 Nov 9;e071707. doi: 10.1136/bmj-2022-071707 36351667 PMC9644277

[65] Pichini A, Ahmed A, Patch C, Bick D, Leblond M, Kasperaviciute D, et al. Developing a National Newborn Genomes Program: An Approach Driven by Ethics, Engagement and Co-design. Front Genet. 2022;13:866168. doi: 10.3389/fgene.2022.866168 35711926 PMC9195613

[66] NHS England. NHS Genomic Medicine Service [Internet]. [cited 2023 Feb 23]. Available from: https://www.england.nhs.uk/genomics/

[67] Killcoyne S, Gregson E, Wedge DC, Woodcock DJ, Eldridge MD, de la Rue R, et al. Genomic copy number predicts esophageal cancer years before transformation. Nat Med. 2020 Nov;26(11):1726–32. doi: 10.1038/s41591-020-1033-y 32895572 PMC7116403

[68] Wolfson M, Gribble S, Pashayan N, Easton DF, Antoniou AC, Lee A, et al. Potential of polygenic risk scores for improving population estimates of women’s breast cancer genetic risks. Genetics in Medicine. 2021 Nov;23(11):2114–21. doi: 10.1038/s41436-021-01258-y 34230637 PMC8553614

