# Supplementary material for: Ethical and legal implications of implementing risk algorithms for early detection and screening for oesophageal cancer, now and in the future
Source: PLoS One. 2023 Oct 30;18(10):e0293576. doi: 10.1371/journal.pone.0293576 (PMC10615292; doi:10.1371/journal.pone.0293576)
Supplement: S1 File — (DOCX) [file pone.0293576.s001.docx]

Workshop Report on Adopting a Risk Tool for Stratification and Predictive Prevention of Oesophageal Cancer

PHG Foundation supported by Cancer Research UK & Innovate UK as part of Project DELTA

**
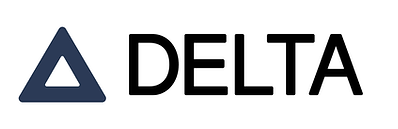
**

Authors

Tanya Brigden, Colin Mitchell, Elizabeth Redrup Hill and Alison Hall

Funding

This work was supported by Cancer Research UK & Innovate UK [grant number 41662]. This report has been drafted pursuant to Sub-Deliverable 1.4.2 of this Grant.

Acknowledgements

We are extremely grateful to our speakers and workshop participants for generously providing their time and expert input. In particular, we are grateful to Professor Rebecca Fitzgerald and Professor Julia Hippisley-Cox for their insights into CanPredict and Cytosponge™, and for their valuable comments on this workshop report. We are also grateful to our other Project DELTA collaborators for their feedback on this research as it progressed, and to the funders for their support.

Disclaimer

The following report is intended to provide general information and understanding of the law. The report should not be considered legal advice, nor used as a substitute for seeking qualified legal advice.

URLs in this report were correct as of March 2023

This report is available from www.phgfoundation.org

Published by PHG Foundation 2 Worts Causeway, Cambridge, CB1 8RN, UK +44 (0)1223 761900

© PHG Foundation

Correspondence to:

intelligence@phgfoundation.org

How to reference this report:

Brigden T, Mitchell C, Redrup Hill E, Hall A. Workshop report on adopting a risk tool for stratification and predictive prevention of oesophageal cancer. PHG Foundation supported by Cancer Research UK & Innovate UK as part of Project DELTA. 2023.

PHG Foundation is an exempt charity under the Charities Act 2011 and is regulated by HEFCE as a connected institution of the University of Cambridge. We are also a registered company No. 5823194, working to achieve better health through the responsible and evidence based application of biomedical science

**Contents**

[1. Introduction 4](#_Toc143082077)

[2. Aims 5](#_Toc143082078)

[3. Background 6](#_Toc143082079)

[How could risk algorithms be used in cancer detection? 6](#_Toc143082080)

[How are risk prediction tools currently used in primary care? 6](#_Toc143082081)

[Development of a risk algorithm for oesophageal cancer for use in primary care 7](#_Toc143082082)

[4. Methods 7](#_Toc143082083)

[5. Key themes and findings 9](#_Toc143082084)

[5.1 Supporting clinicians to use cancer risk prediction algorithms 10](#_Toc143082085)

[5.2 Transparency and risk communication 11](#_Toc143082086)

[5.3 Bias, equity and fairness 14](#_Toc143082087)

[5.4 Managing resources 16](#_Toc143082088)

[5.5 Avoiding exceptionalism 16](#_Toc143082089)

[5.6 Looking to the future 17](#_Toc143082090)

[6. Conclusions 18](#_Toc143082091)

[Policy considerations 19](#_Toc143082092)

[References and Notes 21](#_Toc143082093)

# 1. Introduction

Recent healthcare policy has placed increasing emphasis on stratifying populations to identify those people who are at greater disease risk, in order to offer them interventions to prevent disease from occurring or to manage disease more effectively. Depending on the condition, this could include more intensive management of those at most risk. Risk prediction tools provide a mechanism for identifying these individuals and facilitating access to appropriate interventions, treatments and management. Cancer risk prediction tools (such as QCancer and RATs) have been developed for this purpose, for use in primary care in England, however they remain an underused resource. Such tools have the potential to improve the early detection of cancer and make the best use of available resources but they also give rise to potential ethical and legal considerations.

On 27th September 2022, the PHG Foundation held a workshop that explored the ethical and legal implications of using a risk tool for risk stratification of oesophageal cancer. This report forms part of Project DELTA, funded by Innovate UK and Cancer Research UK which aims to improve the diagnosis of oesophageal cancer. It is an output from Work Packages 1 and 2 of the project which aim to develop and validate an algorithm aimed at risk-stratifying patients for whom early investigation will be beneficial, and implement this in clinical pathways for Cytosponge™. It is hoped that the use of this algorithm in the form of a risk prediction tool could help to improve early recognition of oesophageal cancers and to reduce overuse of prescription acid-regulating therapies.

This project aims to improve the diagnosis of oesophageal cancer by detecting cell changes in the lower oesophagus which are a precursor to cancer, called Barrett’s oesophagus. These changes are caused by the reflux of acid and bile from the stomach, often resulting in heartburn symptoms. In Western countries, around 10-20% of the adult population are affected by gastro-esophageal reflux disease (GERD)^[[1]](#endnote-1)^ of whom between 1.8% - 7.5% may have Barrett’s oesophagus.^[[2]](#endnote-2)^ Barrett’s oesophagus may also occur in asymptomatic individuals. Early detection of individuals with Barrett’s oesophagus could enable detection of those individuals at highest risk of oesophageal cancer.

# 2. Aims

The aim of Project DELTA^[[3]](#endnote-3)^ is to utilise novel methods (including AI) throughout the patient pathway to:

1. Identify those who may be at increased risk through applying a novel risk-algorithm to electronic health records (Work Package 1)

2. Develop a novel, less invasive, sampling method (the Cytosponge™) to collect cells from the oesophagus (Work Package 2)

3. Apply a novel stain (Trefoil factor 3 (TFF3)) to these cell samples to more accurately identify cellular changes indicating disease (intestinal metaplasia) (Work Package 3)

4. Develop AI tools to support, and in some cases, replace assessment and interpretation of these cellular samples by trained pathologists, to identify relevant cellular changes (e.g. from stratified squamous cells to columnar epithelium containing goblet cells) in order to guide future patient management (Work Package 3)

The PHG Foundation undertook a cross cutting programme of research across these work packages in order to deliver a wide range of outputs focusing on legal/regulatory and ethical issues and challenges associated with the application of a novel risk -algorithm to electronic health records (Work Package 1); developing a novel sampling method, the Cytosponge™(Work Package 2); and developing and using a novel stain to identify cellular changes indicating disease and identifying these changes, using AI tools to supplement the assessment by pathologists (Work Package 3).

This report describes research done by the PHG Foundation to assess the legal/regulatory and ethical challenges associated with implementing a risk tool with the intention of enabling earlier detection of oesophageal cancers and Barrett’s oesophagus. The aims of this research were:

- to identify how a risk tool for risk stratification and predictive prevention could be incorporated in existing pathways
- to identify the ethical and legal implications that may be generated
- to evaluate any additional implications that should be taken into account in the short-medium term by researchers, healthcare professionals and policymakers
- to consider the implications of more speculative uses of the risk algorithm/tool.

# 3. Background

## How could risk algorithms be used in cancer detection?

There are a variety of ways in which risk prediction algorithms could be used for the detection and treatment of oesophageal cancer and Barrett’s oesophagus. Project DELTA focused on two possible applications where the use of risk tools have potential utility:

- Targeted screening in symptomatic patients: By integrating risk information in electronic health records, potentially supplemented by additional information from a face to face encounter with a patient, the risk algorithm/tool could be used to triage patients identifying those at higher risk of oesophageal cancer who could be referred for a Cytosponge^TM^ or endoscopy. Patients with new onset of ‘alarm’ symptoms for cancer requiring immediate investigation (vomiting blood, weight-loss or difficulty swallowing) are excluded. This assessment will generally take place in primary care.
- Surveillance of those with a diagnosis of known Barrett’s oesophagus in order to guide patient management, through informing the nature of the surveillance that should be undertaken (e.g. Cytosponge^TM^ or endoscopy), and the frequency of that intervention.^[[4]](#endnote-4)^ If this decision and intervention involves endoscopy it will generally take place in secondary care settings.

## How are risk prediction tools currently used in primary care?

Statistical formulae have been developed that can be used to calculate the probability that an individual patient will develop a cancer within a specified period. Such cancer risk prediction tools remain an underused resource within primary care in England due to a range of practical and operational reasons.^[[5]](#endnote-5)^ Cancer risk algorithms could support the clinicians’ assessment of patients’ presenting symptoms, and guide decisions about onward referral for investigation or specialist assessment. Patients suspected of cancer are referred by their General Practitioners (GPs) to the 2 week-wait referral pathway^[[6]](#endnote-6)^ but identifying eligible patients is difficult especially where early cancers present with vague, non-specific symptoms that are replicated in many other conditions thus confounding prompt diagnosis.^[[7]](#endnote-7)^

Cancer risk assessment tools, most notably QCancer® and Risk-Assessment Tools (RATs), have been integrated with the electronic health record in some parts of UK primary care and as of 2019, cancer risk tools were available to approximately a third of all primary care practices in the UK, with 18.5% of practices having access to these in electronic form.^[[8]](#endnote-8)^ Even where they are accessible, GP’s may be reluctant to rely on them. Taken together, these factors, and difficulties integrating them into the clinical workflow mean that they are not widely utilised.^[[9]](#endnote-9)^

## Development of a risk algorithm for oesophageal cancer for use in primary care

Primary care electronic records contain a rich diversity of demographic, clinical and medication information which could be used for prediction and prevention. Professor Hippisley-Cox and her team have developed a range of risk prediction algorithms for different conditions including coronary vascular disease. These have utilised a master dataset derived from a coalition of GP practices containing records from around 30 million patients (the QResearch network) and similar methodology. Work Package 1 of Project DELTA aimed to develop an algorithm for estimating the 10-year risk of developing oesophageal cancer, given the absence of such a tool to integrate data from relevant clinical, medical and demographic information contained in primary care electronic records. Using a dataset of around 17 million eligible patients, and linking to cancer registry data, Office of National Statistics death registry and hospital episode statistics data (HES), the team generated statistics on the incidence of oesophageal cancer. They then evaluated the contribution of established and novel risk factors^[[10]](#endnote-10)^ before developing a list of predictors that were incorporated in the final algorithms. Key risk factors include medication used to relieve the symptoms of gastroesophageal reflux disease including prescriptions for proton pump inhibitors, and H2 blockers which both reduce the amount of stomach acid made by glands lining the stomach in different ways. These drugs are commonly prescribed within primary care but are also available over the counter.

Hippisley-Cox and colleagues estimate that identifying the top 25% of patients at highest risk would capture 76% of oesophageal cancers that would develop over the next 10 years.^[[11]](#endnote-11)^

Given these potential opportunities, our aim was to identify and evaluate the ethical and legal/regulatory issues that might be raised.

# 4. Methods

A multidisciplinary expert workshop was designed to evaluate the ethical and legal factors that might be generated by the use of a risk tool for risk stratification and predictive prevention of Barrett’s oesophagus as an important risk factor for oesophageal cancer. Two specific contexts were explored: symptomatic referral of patients who present to their GP with symptoms, and surveillance of those with a known diagnosis of Barrett’s oesophagus. Participants also considered more speculative future uses. Participants were provided with a briefing note prior to the workshop which described the epidemiology of Barrett’s oesophagus, the aims and objectives of Project DELTA, an introduction to the novel risk-algorithm (CanPredict) which can be used to identify people at increased risk of oesophageal cancer from electronic health records, and some broad ethical and legal considerations to reflect on in advance of the workshop. Participants were recruited to this workshop on a purposive basis with the objective of ensuring representation from key stakeholder groups including software/algorithm/risk tool developers; ethical and legal experts; clinicians; patient representatives; collaborators from Project DELTA and representatives from professional organisations.

*Figure 1 Stakeholder workshop aims and objectives*

| **Workshop aims and objectives** |
| --- |
| 1. To explore how a risk tool for risk stratification and predictive prevention might be incorporated together with Cytosponge^TM^ in existing pathways for the early detection of Barrett’s oesophagus and oesophageal cancer 2. To identify and evaluate ethical and legal factors that may be generated by the use of the risk tool in each of these contexts 3. To consider how these factors may impact design and implementation of the risk tool 4. To consider the implications of more speculative uses of the risk algorithm/risk tool, such as in asymptomatic patients, and the impacts of automating the process of data mining using artificial intelligence. |

The multidisciplinary workshop was held via a virtual platform between 9.30-12.00 on 27th September 2022, attended by twenty one participants (including five researchers from PHG Foundation). Two initial presentations from DELTA collaborators described the context for, and process of, developing the risk algorithm for oesophageal cancer, and for developing nurse-led clinics to administer Cytosponge^TM^. The workshop then proceeded as semi-structured plenary discussion divided into two sessions, conducted under Chatham House Rules, with a recording of the workshop being taken for note-taking.

The focus of the first session was on exploring the ethical and legal considerations around the implementation of the risk tool into existing pathways (e.g. for symptomatic referrals and Barrett’s surveillance.) The questions put to participants were the following:

1. Are there are any particularly pertinent legal and ethical considerations that stand out to you when thinking about the use of risk algorithms for symptomatic patients?
2. How do these differ when thinking about:
   - - 1. Deployment in person
       2. Deployment without face-to-face contact
3. Turning to the surveillance pathway where patients already have a diagnosis of Barrett’s oesophagus, are the proposed ethical and legal implications of using the risk algorithm substantially different?

The second session focused on future more speculative uses of the risk algorithm, the potential for the involvement of AI for data mining, and the implications of these developments. Participants were asked to reflect upon the following scenarios:

1. Screening asymptomatic individuals through batch processing risk stratification. This could take the form of
   - Stratification within primary care using GP records
   - Stratification using centralised records as part of a wider screening programme, for example under the auspices of the National Screening Committee
2. The possible direct-to consumer use of the risk tool, through an app or webpage, without oversight from a healthcare professional
3. The potential use of AI in the future (either fully or partially) for data mining, and whether this raises any new considerations

# 5. Key themes and findings

The discussions in the workshop raised many interesting ethical and legal considerations. These have been collated and synthesised below to provide an overview of the key findings, under the themes of supporting clinicians to use cancer risk prediction algorithms; transparency and risk communication; bias, equity and fairness; managing resources; and avoiding exceptionalism.

## 5.1 Supporting clinicians to use cancer risk prediction algorithms

*“We talk a lot about patients buying into risk stratification tools and understanding it, but I think clinicians need to buy into it as well. Clinicians need to understand the mechanisms behind why we have made these decisions and what we factored into it”*

It was acknowledged that support from clinicians is crucial in order for the risk tool to be implemented effectively. Historically, primary care practitioners have demonstrated some reluctance around introducing cancer risk algorithms due to a number of factors including the potential increase in workload that might result, concerns around liability, and lack of supportive activities accompanying implementation. It was suggested that equipping clinicians with information about the algorithm (such as its derivation, validation and accuracy) and the factors contributing to the risk result may contribute to clinician’s willingness to use the tool.

*“GPs have the capacity to refer outside the NG12 and the algorithm-worked-out referral criteria, and GPs will always have the ability to refer any patient that they are worried about regardless of algorithms and other guidelines”*

*“We can have guidance and guidelines. I think there is a danger where they become so rigid that they become restrictions on the ability to deliver individually tailored care where there are risk factors and presentations, or indeed patient concerns, which are understandable and legitimate, and give rise to a reason to deviate from guidance.”*

Recognising that the risk tool is intended to support, rather than override clinical decision-making was seen to be important, with participants commenting that GPs have the capacity to refer outside of the NICE NG12 suspected cancer referral guidance and that the same flexibility would apply to any recommendations made by the algorithm. It was acknowledged that this can in some instances lead to unfairness, as GPs make different decisions based upon their experience. For example a GP who has seen a patient die in their 20’s of oesophageal adenocarcinoma with no previous symptoms may have a lower threshold for referral than one who has not. It was highlighted that although the risk tool is intended to support rather than override clinical decision-making, it may improve the consistency of referral decisions.

“*The potential liability which accrues to a physician or organisation which has the information about increased risk, but doesn’t act upon it, creating a potential future liability under tort for negligence and where that might sit, is important where we have an already overstretched primary care system”*

Participants noted that when considering clinician support for the risk tool, liability might act as a barrier to uptake. They expressed concern about the potential to generate more risk information requiring intervention, and which would inevitably lead to an increase in the number of referrals and a greater workload for already overstretched primary care professionals. The addition of another risk element which requires intervention and action should be carefully considered and reassurance is needed about how conflicts between clinical support tools and professional judgement might be managed.

## 5.2 Transparency and risk communication

*“If you have sat down with your doctor… you are in a decision-making environment and the doctor can explain ’these are the inputs, this is why we are considering them and this is why you are at higher (or lower) risk and these are the options that are available to you now.’ That is a very different thing from suddenly being referred on the basis of an algorithm that you don’t know is being applied… These are two different scenarios in terms of the amount of information that you as a patient are having about what is happening to you.”*

When considering the use of CanPredict and the degree of information that should be provided to patients, workshop participants agreed that there is an important distinction between the use of a risk tool in a context where a symptomatic patient presents to primary care, and the application of the algorithm to a GP practice database to identify high risk patients who are then invited to have a Cytosponge^TM^ test, without them seeking advice. In the former context, the patient is able to benefit from face-to-face risk communication in an environment where a clinician can explain the reasons behind a risk result, and the patient can ask questions. In the latter, the patient does not have the opportunity to engage in a face-to-face discussion with their clinician about the risk-stratification, unless they deliberately seek this out, and the leaflet accompanying the invitation letter may be the only information that they are provided. It was suggested that any information leaflet should explain why the patient is being contacted, the implications of testing/declining testing and acknowledge any uncertainties that may arise. Participants cited existing screening and early detection programmes from which lessons may be learned, including the NHS Health Check programme and Bowel Cancer Screening programme. Language and framing is key to effective communication, and there was some discussion around striking the balance between informing and persuading. The example of the Targeted Lung Health Check programme was raised, which invites people between 55 years and 75 years old that have ever smoked for a free lung health check to identify those most at risk of lung cancer. The reframing of lung cancer screening to a ‘targeted lung health check’ may appear less intimidating to patients, and it was suggested that a similar approach could be adopted for the oesophagus, particularly due to the fact that the screening invitation is for the purpose of detecting Barrett’s oesophagus, a precursor to oesophageal cancer, rather than oesophageal cancer itself. Additional strategies to improve communication might include incorporating a link to a video showing a Cytosponge^TM^ test being performed, so that patients have realistic expectations of the test process.

Additionally, participants noted that good communication (although important) isn’t sufficient in this context, as the act of inviting an individual signals that testing is of benefit to them, undermining the expectation that they can and will act autonomously. It was argued that the ethical obligation is on the provider to ensure that the tests are not harming people in the population who are being approached with an invitation for an intervention.

*“Once you start inviting people, you need to worry more about ‘do no harm’ than autonomy.”*

This is reflected in the debate around breast cancer screening as, despite being a cost effective programme, it has been controversial for many years due to the cohort of women for whom the benefits are unlikely to outweigh the harms and who some believe should not be invited for screening at all. Therefore, where the health system is asking patients to make a decision, rather than simply to comply with a request, this needs to be clear in the language used in the invitation letter so as to frame the patient's expectations.

*“It is a real challenge, very often, for people to understand the background risk, the relative increased risk and the absolute increased risk when it comes to making an informed decision …So there are challenges about the public understanding of risk.. which has to sit at the heart of any informed decision-making with individual patients based on a risk algorithm”*

Participants noted that communicating risk is a familiar challenge in a primary care setting, and that patients often have difficulty understanding relative and absolute risks. They remarked that there is a strong body of evidence to draw on regarding best practice for explaining risk results and the uncertainties around them, and that this risk communication process is vital because it sits at the heart of any informed decision-making.

It is important to be able to explain not only the patient’s result but also how it was calculated and why a recommendation has been made. Participants suggested that one example of this is the PREDICT^[[12]](#endnote-12)^ online tool for breast cancer, which has different layers of explanation so that information provision can be tailored to the preferences of the patient. On the webpage, patients are able to see what the risk factors are, why they were chosen, and should they wish to, the relative risks of each of those factors and even the actual algorithm. This satisfies the legal requirement to provide information under the GDPR in ways that facilitate patient understanding, thus meeting additional legal and ethical obligations for transparency.

*“The discussion about communicating the limitations of the algorithm, including the dataset that it was based on and any potential biases that arise from that, is a really important one”*

Workshop participants highlighted the imperative to be clear where results may be less accurate due to the scarcity of that population subgroup in the training datasets or dataset from which the algorithm was derived. For example there is likely to be very few patients with oesophageal cancer in their 20’s within the training dataset, and therefore the algorithm may underestimate risk in younger patients. In instances where the algorithm may perform less reliably, communication with the patient should be very direct, rather than couched in academic language, to make it clear that limitations in the data lead to greater uncertainty in the results for this group.

“*There is a perception that endoscopy is a perfect test – it’s not. There is a documented miss rate for cancer in patients who have endoscopy, which can be as high as 10-15%. That factors into patient acceptability.”*

Participants raised the challenge of patient acceptability, both in the use of a risk tool but also in the context of the entire testing pathway. There is a challenge in offering the Cytosponge^TM^ instead of, or as a complement to, endoscopy, to patients who have been using endoscopies for surveillance for years and believe it to be the gold standard. Facilitating the shift from concern that testing is being withdrawn, to the understanding that Cytosponge^TM^ could be the preferable option for some groups of patients, will be challenging, and will require clear communication around the purpose and reasons for the shift in their risk management.

## 5.3 Bias, equity and fairness

*“With the modelling process you have to be aware of the limitations of the data, its coverage and whether it is representative”*

Ensuring that the training dataset used for development of an algorithm is representative of the population to which it will be applied, is vital to promote equitable and effective care. Health predictions may be systematically worse for underrepresented groups, than for well represented groups. Workshop participants noted that the QResearch dataset is comprehensive (as over 99% of the general population are registered with a GP, and over 80% have self-reported ethnicity). This has been linked to data from hospitals and the cancer registry, and so the group of patients with oesophageal cancer that present in emergency care, rather than through primary care referrals, are also captured by the algorithm. However, more challenging to account for are the subset of individuals who self-medicate with over-the-counter proton pump inhibitors (PPIs), as in some instances there would be no record of medication use on their EHR. These patients may have a different risk profile and could have their risk misestimated.

*“There is a difference where the algorithm is being applied without talking to people [i.e. batch processing at a GP practice level, as opposed to symptomatic patients presenting in primary care]… that may in a way be more equal.. but if you have a conversation about whether someone’s happy to have an algorithm make a decision about them and their care, if they are not happy then what happens? What happens to people who don’t want that risk assessment?”*

During discussion, challenges around mitigating social inequalities were raised. Some participants were concerned that if the risk tool were to be used during GP appointments initiated by symptomatic patients (rather than applied to all EHR records held by the GP practice to identify those at high risk) then individuals who have not sought guidance or medication from their GP, will potentially be disadvantaged by not being eligible for targeted screening. We know that there are inequalities in how people access treatments, with some patients more likely to (and more able to) engage with primary care than others. Additionally, if the algorithm is trained on data that excludes patients who have not accessed treatment, then that bias could be replicated in future applications of the algorithm.

*“A big concern for public acceptability are notions of perceived fairness. Why is she getting the test and not me?.. Why am I not going straight to endoscopy?… Explainability is also important for resolving those concerns, particularly to the extent that categories used for the risk prediction tool relate to socially salient categories around sex, race, ethnicity, or anything where there is a perceived, and perhaps real, issue of fairness... Everyone might understand what the risks involved are but may still feel uncomfortable about the notion that they are being excluded or other people are being excluded on these bases.”*

Participants raised the notion that *perceptions* of fairness and bias amongst patients and publics are also relevant. Equity and fairness are not easy to measure with the tools available, and measures such as cost effectiveness provide no insight into *who* is receiving the benefit and who is suffering the cost. The example of bowel cancer screening was raised, as although there are more cases of colorectal cancer in men than women, at an earlier age, and the incidence of colorectal cancer related mortality is higher in men^[[13]](#endnote-13)^, there has been pushback against the idea of setting different age thresholds for screening men and women. What if you are a woman whose cancer would have been picked up by screening that you would have been eligible for if you were a man? Although arguably, having different screening approaches for men and women would be more equitable, discomfort around differences in treatment based on socially salient categories could impact perceptions of fairness. It was suggested that being as clear as possible about, first, the balance of benefits and harms for an individual to have the test, and second, why that individual is being offered testing over someone else, might help to alleviate some of this concern and maximise uptake.

*“If you are using protected characteristics in an algorithm which means that some people go to the top of the queue and others are necessarily sliding down that queue, you have to have a defensible basis for doing it… but you can’t just vaccinate everyone immediately because that is just not possible to do, you have to have some sort of queue. So there is no perfect solution.”*

Drawing on experiences of the Covid-19 pandemic, participants noted that prioritising some patients for vaccination inevitably and necessarily deprioritised others, potentially leading to perceptions of unfairness. They acknowledged the differences between the Covid-19 context, where the entire population was eventually offered the intervention, and the Project DELTA context, where only those at high risk receive an intervention. However, in both scenarios an algorithm is used to stratify individuals on the basis of a number of risk factors, including protected characteristics. Widespread understanding that prioritising those at highest absolute risk for interventions may lead to the greatest population benefit has been communicated extensively through the work done by the pandemic vaccination programme, and may help to increase acceptance and understanding of risk stratification.

## 5.4 Managing resources

*“How do you scale up the use of algorithms in combination with Cytosponge and ensure that patients are adequately informed by potential risks and benefits given the restricted resources that we have at present?”*

There was discussion around the challenges of scaling up the use of the algorithm to identify individuals at increased risk of oesophageal cancer, which would inevitably lead to greater numbers in need of testing, and the impact that this would have on an already overstretched health system. Delivering Cytosponge^TM^ at scale will require more healthcare professionals to perform tests (as they cannot be self-administered), infrastructure to conduct analysis on the samples, and may lead to more patients making contact with primary care.

On the other hand, ruling out some patients and being able to prioritise those who are in need of endoscopy may relieve pressure on endoscopists who are currently understaffed leading to long wait times. Indeed, during the Covid-19 pandemic, Cytosponge^TM^ had been used for surveillance of patients with known Barrett’s oesophagus, on an exceptional basis, as an alternative to endoscopy, when the harms associated with endoscopy (unacceptable risk of aerosol production and potentially Covid-19 transmission and the need for extensive infection control measures) were considered to outweigh the potential benefits. Restricting the numbers and conditions in which endoscopies could take place had resulted in bottlenecks for patients, with very small numbers of endoscopies being done. During this period, the use of Cytosponge^TM^ as a replacement for endoscopy offered significant comparative benefits for this group of patients with known Barrett’s oesophagus. However, it was acknowledged that, in normal conditions, widening the scope of targeted screening for those without known Barrett’s oesophagus at an earlier stage of the patient pathway would require more resources, for screening using Cytosponge^TM^ and downstream increases in numbers of patients referred for endoscopy.

## 5.5 Avoiding exceptionalism

*“We have been using risk stratification techniques in our guidelines in secondary care for Barrett’s surveillance for quite a long time… The use of an algorithm isn’t really new”*

*“This is just helpfully putting some more information in the hands of patients and clinicians who are making these decisions every day anyway and it's an entirely easily explicable risk algorithm”*

*​​”We probably shouldn’t get hung up on the fact that it's an algorithm. We use algorithms all the time.”*

Workshop participants were wary of the possibility of exceptionalism when using a risk tool in this context. They felt that it was easy to get caught up in the language of ‘algorithms’, but that the risk model has applied numbers to risk factors that are already known (and used), many of which will be unsurprising to patients.

It was argued that risk stratification techniques are already employed for Barrett’s oesophagus surveillance. Clinicians draw on risk factors such as the length of the Barrett’s segment, and the patient’s age and fitness levels, to ascertain the frequency of surveillance interventions necessary. Risk prediction algorithms are also used across many other medical specialties, and are increasingly embedded in routine clinical practice. Therefore, participants agreed that it was unhelpful and inaccurate to regard the proposed use of an algorithm/risk tool for targeted screening for oesophageal cancer, as exceptional.

## 5.6 Looking to the future

*“If someone had not attended a GP or had anything recorded, the algorithm would tend to underestimate those risks.”*

Workshop participants provided some examples of how existing screening could be expanded in future. This included providing more complete coverage of those potentially at risk, such as those who were not enrolled with a GP, or who were enrolled but had not reported relevant clinical symptoms. However, these issues were discussed more in terms of addressing systematic biases in the data (such as underestimating the risks for this group of individuals) than in terms of recommendations about changes in practice.

*“Self-medicators would be under risked by the algorithm, as taking over-the-counter proton pump inhibitors would not be on a GP’s system. [The] issue that self-medicators [are] a totally unknown group and off radar [is] a bigger public health issue than the algorithm.”*

Similarly, including over the counter medications as an additional risk factor, was raised as a means of extending the coverage of the algorithm beyond electronic records, but this was framed in terms of a more fundamental issue about the generation of evidence in public health policy.

*“If algorithms are used to make decisions about resources in the NHS.. private health care.. undermines the credibility of the algorithm if used in the NHS.”*

Although there was enthusiasm for future expansion of screening programmes, there was concern that publicly funded resources are limited, and that if a risk prediction algorithm is used to inform the allocation of resources within the NHS (including further diagnosis and treatment) that the credibility of the algorithm is potentially undermined by a private health care service which allows access to diagnosis on demand through privately funded health care, and not based on risk.

Machine learning could provide a way of managing increased demand. However, it would be important to compare the performance of any risk prediction algorithm against existing literature as a way of identifying and addressing potential limitations in the data, rather than passing on ‘black box’ results without proper validation.

# 6. Conclusions

This workshop has highlighted that risk prediction algorithms such as CanPredict provide an opportunity to enable earlier detection and screening for oesophageal cancer, through stratifying those patients who have most to gain from onward referral for further investigation. However, its use raises ethical and legal considerations, many of which depend to some extent on how the risk tool is deployed i.e. whether as part of a face-to-face encounter, or systematically applied to an NHS primary care database, or for surveillance of those with a diagnosis of Barrett’s oesophagus. Much of the discussion during the workshop related to ethically relevant aspects of these different approaches e.g. risk assessment in symptomatic vs asymptomatic patients, face-to-face vs written forms of communication. Putting strategies in place to ensure that the tool supports clinician’s work processes was seen to be key, and one approach for doing so is through the provision of support and training to build understanding around, and trust in, the risk tool and the algorithm underpinning it. Emphasising the role of the risk tool as a clinical support tool (rather than a decision-making tool) was also raised on a number of occasions, to reassure clinicians that they have the flexibility to deliver individually tailored care that may deviate from guidance (or the result generated by the tool). Reinforcing this with guidance around managing conflicts between the risk tool and professional judgement may alleviate concerns around liability.

Reducing bias and promoting equity lie at the core of ethical implementation of the risk tool. Relying on a representative database such as QResearch for training the risk prediction algorithm should increase confidence in the accuracy of the stratification process. Interestingly, public *perceptions* of fairness were also identified as an important consideration, exacerbated by the many complex risk factors that contribute to the stratification and which may lead to confusion around why some individuals are prioritised over others.

Transparency and a focus on effective communication is vital to mitigate this and build trustworthiness. However, throughout discussion it was made clear that whilst CanPredict is a novel risk algorithm that will be implemented as part of a novel pathway (which includes Cytosponge^TM^), the use of risk algorithms in clinical care is not itself novel and does not necessarily warrant exceptional treatment. As such, lessons can be learned, both from the use of risk algorithms deployed in other areas of clinical care (such as QRISK2 in the NHS Health Check programme), as well as from other population level screening programmes that invite asymptomatic individuals for testing/screening following some form of risk stratification.

## Policy considerations

Novel pathways incorporating risk prediction algorithms (CanPredict) combined with a non-invasive, low-cost sampling device (Cytosponge^TM^), hold considerable promise. Our findings suggest the following considerations for policymakers, regulators and developers in order to maximise the benefits that can be afforded by implementing a risk tool into early detection pathways for oesophageal cancer.

1. Building on the current body of evidence, more research is needed to establish the quantity and types of information that clinicians and patients need (and want) to know about the development and use of the risk algorithm, in order to build understanding, trustworthiness, and facilitate realistic patient expectations. Equally important is identifying effective methods to communicate that information to patients and healthcare professionals.
2. Consideration must be given to the impact that the use of a CanPredict, in combination with Cytosponge^TM^, will have on the existing processes and pathways. This may range from necessary operational changes such as integration of CanPredict into electronic health records systems, to strategies to extend screening and diagnostic tests to the increased numbers of high risk patients that are likely to be identified through the systematic use of a risk tool.
3. Strategies to promote equitable access should be prioritised. These might include actively applying the risk algorithm to NHS databases rather than relying on patients to engage with primary care themselves. Identifying high risk individuals in lower socio-economic groups and inviting them for Cytosponge^TM^ testing may contribute to efforts to narrow healthcare inequalities and meet wider policy goals such as those laid out by Core20PLUS5.
4. Guidance to clarify how healthcare professionals should manage conflicts between clinical support tools and professional judgement is required to resolve concerns around liability, a key barrier to uptake.
5. There is potential for risk prediction algorithms to incorporate new risk factors or novel methodology involving machine learning/AI in future. If these are adopted, developers and regulators should consider the nature and balance of potential benefits and harms that may arise, in order to ensure that greater accuracy of risk stratification is not achieved at the expense of declining transparency, which could undermine confidence in these clinical support tools.

# References and Notes

1. El-Serag HB, Sweet S, Winchester CC, Dent J. Update on the epidemiology of gastro-oesophageal reflux disease: a systematic review. *BMJ Gut*. 2014;63:871-880. Doi: http://dx.doi.org/10.1136/gutjnl-2012-304269 [↑](#endnote-ref-1)
2. Fan X, Synder N. Prevalence of Barrett’s Esophagus in Patients with or without GERD Symptoms: Role of Race, Age, and Gender. *Digestive Diseases and Sciences*. 2009;54:572-577. DOI <https://doi.org/10.1007/s10620-008-0395-7>; Rex DK, Cummings OW, Shaw M et al. Screening for Barrett's esophagus in colonoscopy patients with and without heartburn. *Gastroenterology*. 2003; 125(6):1670-1677. DOI 10.1053/j.gastro.2003.09.030; Herrera Elizondo JL, Monreal Robles R, Garcia Compean D et al., Prevalence of Barrett's esophagus: An observational study from a gastroenterology clinic. *Revista de gastroenterologia de Mexico*. 2017;82(4):296-300. DOI 10.1016/j.rgmx.2017.01.006. [↑](#endnote-ref-2)
3. Project DELTA. Available at: https://www.deltaproject.org/. Accessed 09 February 2023. [↑](#endnote-ref-3)
4. The British Society for Gastroenterology recommends that patients with Barrett’s oesophagus should be monitored by endoscopy depending on the length of the segment (Patients with Barrett’s oesophagus should receive endoscopic surveillance every 3–5 years (for patients with Barrett’s oesophagus with intestinal metaplasia shorter than 3 cm), or every 2–3 years (patients with segments of 3 cm or longer). See Fitzgerald RC, di Pietro M, Ragunath K*, et al.* British Society of Gastroenterology guidelines on the diagnosis and management of Barrett’s oesophagus. *Gut* 2014;63:7-42. [↑](#endnote-ref-4)
5. Price S, Spencer A, Medina-Lara A, Hamilton W. Availability and use of cancer decision-support tools: a cross-sectional survey of UK primary care. *British Journal of General Practice.* 2019;69(684): e437-e443 [↑](#endnote-ref-5)
6. National Institute for Health and Clinical Excellence. Referral guidelines for suspected cancer. London: National Institute for Health and Clinical Excellence; 2005. [↑](#endnote-ref-6)
7. Sirota M, Kostopoulou O, Round T, Samaranayaka S. Prevalence and alternative explanations influence cancer diagnosis: an experimental study with physicians. *Health Psychology*. 2017;36(5):477-485 [↑](#endnote-ref-7)
8. Price S, Spencer A, Medina-Lara A, Hamilton W. Availability and use of cancer decision-support tools: a cross-sectional survey of UK primary care. *British Journal of General Practice.* 2019;69(684): e437-e443 [↑](#endnote-ref-8)
9. ibid. [↑](#endnote-ref-9)
10. Hippisley-Cox J, Mei W, Fitzgerald R, Coupland C. Development and validation of a novel risk prediction algorithm to estimate 10-year risk of oesophageal cancer in primary care: prospective cohort study and evaluation of performance against two other risk prediction models. *The Lancet Regional Health – Europe.* 2023;32:100700 [↑](#endnote-ref-10)
11. Sirota M, Kostopoulou O, Round T, Samaranayaka S. Prevalence and alternative explanations influence cancer diagnosis: an experimental study with physicians. *Health Psychology*. 2017;36(5):477-485 [↑](#endnote-ref-11)
12. Predict Breast Cancer. Available at: <https://breast.predict.nhs.uk/predict_v1.2.html>. Accessed 29 March 2023 [↑](#endnote-ref-12)
13. Thomas C, Mandrik O, Whyte S, Saunders CL, Griffin SJ, Usher-Smith JA. Should colorectal cancer screening start at different ages for men and women? Cost-effectiveness analysis for a resource-constrained service. *Cancer Rep (Hoboken)*. 2021;4(4):e1344. doi: 10.1002/cnr2.1344


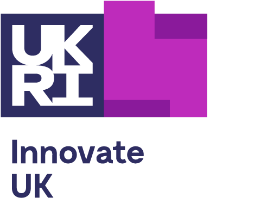

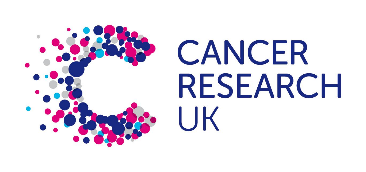
 [↑](#endnote-ref-13)
